# Medicinal chemistry perspectives on anticancer drug design based on clinical applications (2015–2025)

**DOI:** 10.1039/d5ra05472a

**Published:** 2025-10-01

**Authors:** Ahmed A. Al-Karmalawy, Mohamed E. Eissa, Nada A. Ashour, Tarek A. Yousef, Arwa Omar Al Khatib, Samia S. Hawas

**Affiliations:** a Department of Pharmaceutical Chemistry, College of Pharmacy, The University of Mashreq Baghdad 10023 Iraq akarmalawy@horus.edu.eg; b College of Science, Chemistry Department, Imam Mohammad Ibn Saud Islamic University (IMSIU) Riyadh 11623 Saudi Arabia; c Department of Pharmacology and Toxicology, Faculty of Pharmacy, Mansoura National University Gamasa 7731168 Egypt; d Faculty of Pharmacy, Hourani Center for Applied Scientific Research, Al-Ahliyya Amman University Amman Jordan; e Department of Pharmaceutical Chemistry, Faculty of Pharmacy, Horus University-Egypt New Damietta 34518 Egypt

## Abstract

Cancer therapy has undergone a remarkable evolution over the past few decades, driven largely by innovations in medicinal chemistry. This review explores the pivotal role of medicinal chemistry in designing, optimizing, and classifying anticancer agents, from traditional cytotoxics to modern targeted therapies, immunotherapies, and radiotheranostics. The article categorizes FDA-approved anticancer drugs (2015–2025), evaluates their mechanisms of action, structural features, and structure–activity relationships (SAR), and highlights both success stories and challenges in clinical translation. Additionally, it examines withdrawn agents and investigational drugs currently in clinical trials, providing insights into emerging modalities such as PROTACs, antibody–drug conjugates, molecular glues, and AI-driven drug discovery. This synthesis underscores how structure-based drug design, pharmacokinetic modeling, and bioengineering approaches continue to shape the landscape of cancer treatment. Ultimately, medicinal chemistry remains at the heart of the drug development pipeline, offering refined tools for precision oncology and the future of personalized cancer care.

## Abstract 2

Medicinal chemistry perspective on anticancer drug design (2015–2025).

## Graphical abstract

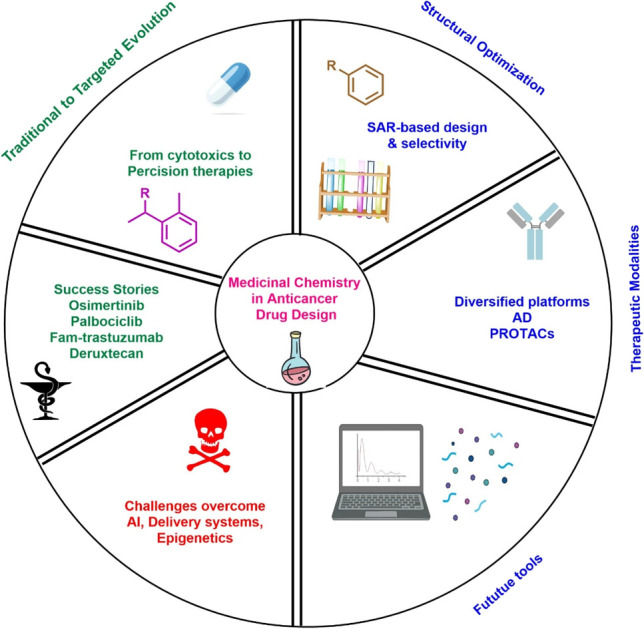

## 1. Introduction

Cancer remains one of the most challenging diseases to treat because of its biological complexity, genetic heterogeneity, and ability to evade conventional therapies.^1,2^ Though chemotherapy was once considered the cornerstone of cancer treatment, its non-selective nature often resulted in significant toxicity and limited long-term success.^3,4^ However, the treatment paradigm in oncology has shifted dramatically over the past few decades due to our growing understanding of cancer biology, drug mechanisms, and specifically, developments in medicinal chemistry.^5,6^

Ancient Egyptian civilizations gave the earliest evidence of cancer treatment (circa 2600 BCE), which was limited to surgical excision or cauterization.^7^ In the 19th century, a pivotal shift occurred when Munir *et al.* presented the concept of stimulating the immune system to fight cancer using bacterial toxins as a pioneer in modern immunotherapy.^8^ The late 20th century witnessed a transition from broadly cytotoxic agents to more selective and mechanism-driven therapies upon the approval of imatinib mesylate in 2001, an incredible moment in precision oncology. It was designed to inhibit the fusion gene resulting from a chromosomal translocation (BCR-ABL) tyrosine kinase fusion protein in chronic myeloid leukemia (CML) and imatinib established the paradigm of structure-based drug design and opened the door for an entire class of kinase inhibitors.^9,10^ Since then, medicinal chemists have engineered inhibitors targeting epidermal growth factor receptor (EGFR), anaplastic lymphoma kinase (ALK), B-Raf proto-oncogene, serine/threonine kinase (BRAF), and other kinases with unique selectivity and clinical impact.^11^

**Fig. 1:**
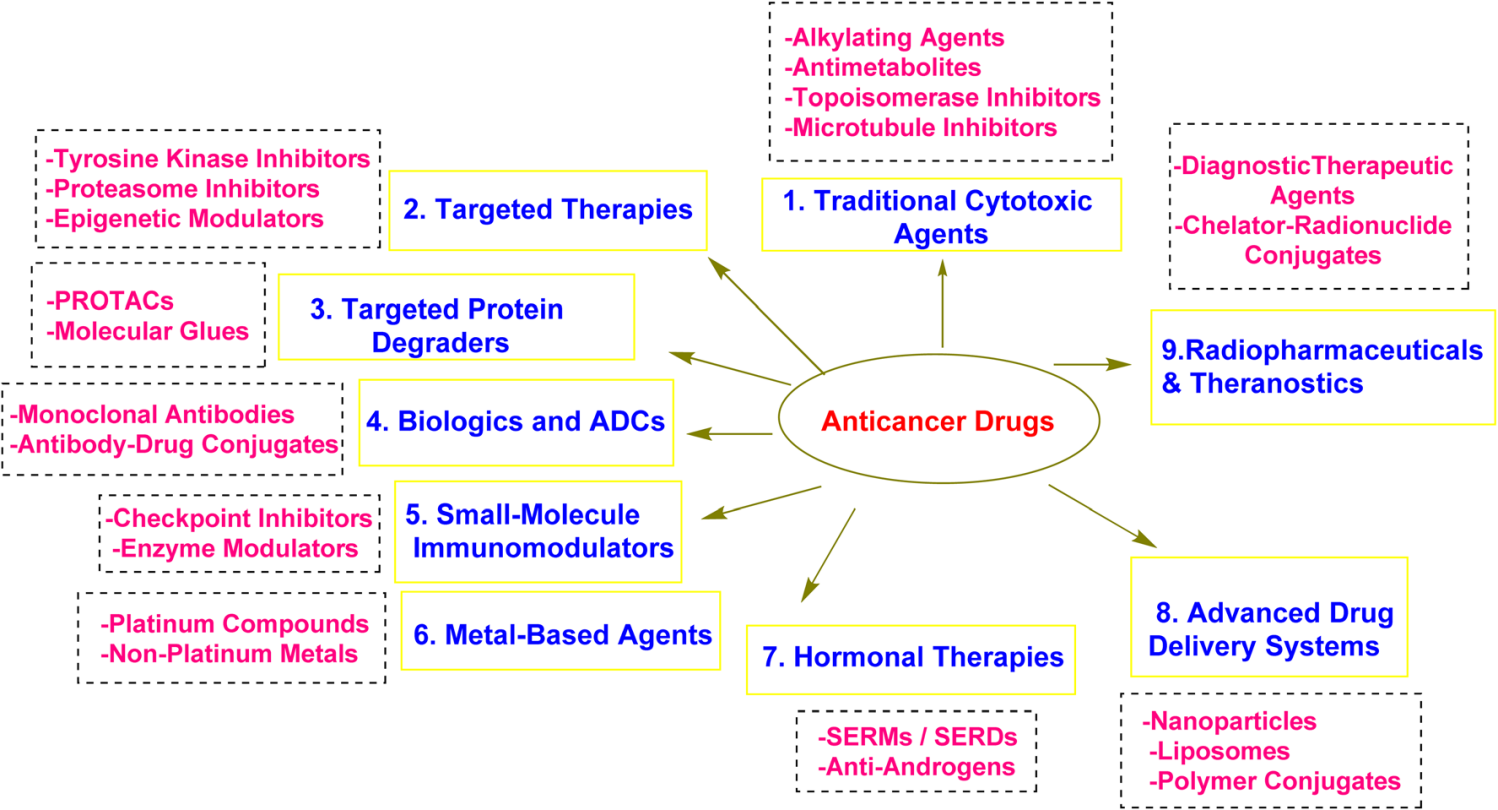
Classifications of anticancer drugs.

The field of anticancer agents has evolved remarkably over the past decades, directed by advances in medicinal chemistry that have enabled the design of increasingly selective and effective therapeutic modalities.^12,13^ These agents can be broadly categorized based on their mechanism of action, molecular targets, and chemical structures, each reflecting different phases in the history and innovation of cancer therapy (as seen in Fig. 1).^14^

Traditional cytotoxic agents represent the earliest class of anticancer drugs and remain essential in many treatment regimens.^15,16^ These include alkylating agents, such as cyclophosphamide and melphalan, which function by forming covalent bonds with deoxyribonucleic acid (DNA), causing crosslinking and cellular apoptosis.^17,18^ Antimetabolites like 5-fluorouracil and methotrexate mimic natural substrates, interfering with DNA and ribonucleic acid (RNA) synthesis.^19^ Agents that target DNA topology, such as topoisomerase inhibitors (*e.g.*, doxorubicin and irinotecan), intercalate into DNA and disrupt replication.^20–22^ Microtubule-targeting agents like paclitaxel and vincristine interfere with mitotic spindle formation, halting cell division.^23^ Despite their efficacy, these agents often suffer from limited selectivity, leading to significant off-target toxicity.^24^

The rise of targeted therapies marked a paradigm shift in oncology, enabling the development of small molecules that specifically inhibit oncogenic drivers.^25–28^ Among the most successful are tyrosine kinase inhibitors (TKIs), such as imatinib for BCR-ABL-positive leukemia and osimertinib for EGFR-mutant lung cancers. These drugs are typically designed to occupy the adenosine triphosphate (ATP)-binding pocket of kinases, with extensive structure–activity relationship (SAR) studies guiding their optimization for potency, selectivity, and pharmacokinetics.^9,29,30^ Proteasome inhibitors, including bortezomib and carfilzomib, represent another targeted class that induces proteotoxic stress by disrupting protein degradation pathways.^31,32^ Similarly, targeting epigenetic regulators such as histone deacetylases (HDACs) and DNA methyltransferases (DNMTs) has opened new avenues in treating hematologic malignancies and solid tumors.^33^ These epigenetic drugs are chemically engineered to interfere with the enzymatic activity responsible for gene silencing in cancer.^34^

In addition to inhibition, the targeted breakdown of disease-causing proteins has been made possible using medicinal chemistry.^35^ Proteolysis-targeting chimeras (PROTACs) are a notable innovation, designed as bifunctional molecules that bring target proteins into proximity with E3 ubiquitin ligases, promoting their degradation by the proteasome.^36^ Molecular glues, which stabilize protein–protein interactions between a target and an E3 ligase, represent another strategy to eliminate previously “undruggable” proteins.^37^ These emerging modalities offer advantages in terms of potency, selectivity, and the ability to address resistance.^38^

Biologics and antibody–drug conjugates (ADCs) represent a fusion of biological targeting and medicinal chemistry.^39^ ADCs such as brentuximab vedotin and trastuzumab deruxtecan combine the selectivity of monoclonal antibodies with potent cytotoxic payloads, linked *via* carefully engineered chemical linkers.^40^ The success of ADCs underscores the importance of linker chemistry, payload selection, and the optimization of drug-to-antibody ratios to balance efficacy and safety.^41^

The field of immunotherapy is dominated by biologics; however, medicinal chemistry is becoming more and more involved in the development of small-molecule immunomodulators.^42^ These compounds aim to modulate immune checkpoints, such as programmed cell death protein 1 (PD-1) and its ligand, programmed death-ligand 1 (PD-L1), or target intracellular enzymes like coactivator-associated arginine methyltransferase 1 (CARM1), which are involved in epigenetic regulation of immune responses.^43^ Unlike monoclonal antibodies, small molecules can offer oral bioavailability, lower production costs, and better tissue penetration, making them attractive candidates for combination therapy.^44^

A re-emerging area of interest involves metal-based anticancer agents.^45^ Although platinum drugs like cisplatin remain essential in clinical oncology, new agents based on metals such as ruthenium and gallium are under development.^46^ These compounds are being engineered with unique redox properties, coordination geometries, and ligand structures to overcome resistance and minimize systemic toxicity.^47^

Advances in drug delivery systems have also been deeply informed by medicinal chemistry.^48–50^ Nanoparticles, liposomes, micelles, and polymer–drug conjugates are increasingly employed to improve drug solubility, stability, and tumor selectivity.^51–53^ The chemical modification of surface ligands and responsive linkers ensures controlled release within the tumor microenvironment.^54^ These innovations enhance therapeutic efficacy while minimizing off-target effects.^55^ Additionally, hormonal therapies remain relevant in hormone-driven cancers.^56^ Agents such as tamoxifen (a selective estrogen receptor modulator) and fulvestrant (a selective estrogen receptor degrader) are chemically tailored to disrupt estrogen receptor signaling.^57,58^ In prostate cancer, anti-androgens like enzalutamide inhibit androgen receptor activation and nuclear translocation, illustrating how subtle structural modifications translate into therapeutic precision.^59^ The integration of radiopharmaceuticals and theranostics is the latest development in the discipline, where medicinal chemistry is crucial to the development of molecules with both therapeutic and diagnostic purposes.^60^ Chelator chemistry and radionuclide conjugation are critical to ensuring selective tumor uptake and minimizing radiation exposure to healthy tissues.^61^ Together, these diverse classes illustrate how medicinal chemistry underpins the innovation, design, and optimization of anticancer drugs across traditional and emerging therapeutic strategies.^62^ From classical cytotoxics to modern targeted degraders, the field continues to evolve with the shared objective of achieving maximum efficacy and safety for patients with cancer.

Radiotheranostics integrate molecular imaging and targeted radionuclide therapy within a single chemical framework, allowing both diagnosis and treatment using the same targeting vector. The medicinal chemistry underpinning these agents centers on three elements: (i) the ligand, typically a peptide, antibody, or small molecule, optimized for high affinity and selective receptor binding; (ii) the chelating moiety, such as 1,4,7,10-tetraazacyclododecane-1,4,7,10-tetraacetic acid (DOTA), 1,4,7-triazacyclononane-1,4,7-triacetic acid (NOTA), or *N*,*N*′-bis[2-hydroxy-5-(carboxyethyl)benzyl]ethylenediamine-*N*,*N*′-diacetic acid (HBED-CC), which ensures stable coordination of radiometals and influences pharmacokinetic behavior; and (iii) the radionuclide, whose emission properties define diagnostic *versus* therapeutic application.^63^ Clinically validated theranostic pairs include ^68Ga/^177Lu-DOTATATE for somatostatin receptor–positive neuroendocrine tumors and ^68Ga/^177Lu-PSMA-617 for prostate cancer, which highlight the seamless transition from imaging to therapy with the same molecular scaffold.^64,65^ More recently, α-emitting isotopes such as ^225Ac and ^213Bi have been introduced to exploit their high linear energy transfer and short tissue penetration, particularly for micrometastatic and resistant disease settings.^66^ Advances in linker and chelator chemistry have further refined biodistribution, improved tumor uptake, and reduced off-target toxicity.^67^ Together, these developments illustrate how radiotheranostics exemplify the convergence of ligand design, chelation chemistry, and isotope selection, opening new frontiers for precision oncology and beyond.^68,69^

Artificial intelligence (AI) has become a transformative driver in medicinal chemistry, shifting optimization strategies beyond classical SAR.^70^ Machine-learning and deep-learning models are increasingly applied to *de novo* molecular design, scaffold hopping, polypharmacology prediction, and property optimization.^71^ Unlike iterative analogue synthesis, AI enables rapid exploration of chemical space by integrating structural, bioactivity, and ADME datasets into predictive models.^72^ A striking example is the GENTRL platform, which generated potent discoidin domain receptor 1 (DDR1) kinase inhibitors *de novo*, followed by experimental validation, demonstrating how generative models can accelerate early discovery.^73^ Broader reviews highlight applications of graph neural networks, reinforcement learning, and active learning loops, which propose novel scaffolds while incorporating synthetic feasibility and experimental feedback.^74^ Importantly, AI methodologies are now also being applied to radiotheranostic design, including optimization of peptide ligands and prediction of isotope distribution patterns, illustrating synergy with other emerging modalities.^75^ While challenges such as data bias and interpretability remain, the trajectory suggests that AI-driven medicinal chemistry will increasingly complement and reshape conventional workflows.^76^

This review aims to provide a comprehensive overview of anticancer drug development from a medicinal chemistry perspective. We will explore molecular design strategies, structure-based innovations, and chemical strategies behind clinically approved and investigational agents, and highlight future directions from artificial intelligence in drug design to new modalities like molecular glues and radiotheranostics. Together, these insights underscore the indispensable role of medicinal chemistry in advancing the fight against cancer.

## 2. Classification of anticancer agents according to their clinical phase

### 2.1. Food and drug administration (FDA)-approved anticancer drugs (2015–2025)

#### 2.1.1. Targeted therapies

##### 2.1.1.1. Tyrosine kinase inhibitors (TKIs)

###### 2.1.1.1.1. Osimertinib (Tagrisso)

Osimertinib is a third-generation, oral EGFR TKI specifically designed to target both sensitizing EGFR mutations (*e.g.*, exon 19 deletions and L858R) and the T790M resistance mutation that often emerges after first-line EGFR TKI therapy in non-small cell lung cancer (NSCLC).^77^ Unlike earlier-generation EGFR inhibitors, osimertinib binds irreversibly to the mutant receptor *via* covalent interaction, offering enhanced potency and selectivity while sparing wild-type EGFR to reduce off-target toxicity. Clinically, osimertinib has demonstrated robust efficacy in patients with T790 M-positive NSCLC, significantly prolonging progression-free and overall survival compared to platinum-based chemotherapy.^78^ The drug exhibits a long half-life (∼48 hours), is metabolized primarily *via* Cytochrome P-450 3A4/5 (CYP3A4/5), and is administered once daily. It received FDA approval in 2015 and later gained expanded indications, including first-line therapy for EGFR-mutated metastatic NSCLC^79^ (Table 1).

**Table 1:** FDA-approved drugs' therapeutic uses, mechanism of action, indication, subclass, and SAR

Drug name	Therapeutic class	Mechanism of action	Indication	Subclass	Structure–activity relationship (SAR)
Osimertinib	Targeted therapy	EGFR T790M inhibitor	NSCLC	Tyrosine kinase inhibitor	Acrylamide warhead: covalent EGFR Cys797; methoxy group: T790M selectivity; aniline: ↓ CYP3A4 metabolism
Tivozanib	Targeted therapy	VEGFR inhibitor	Renal cell carcinoma	Tyrosine kinase inhibitor	Urea linker: H-bond to VEGFR2; chlorophenyl: Hydrophobic moiety; polar chains: selectivity/solubility
Romvimza (vimseltinib)	Targeted therapy	CSF1R inhibitor	Tenosynovial giant cell tumor	Tyrosine kinase inhibitor	Amide linkers for binding; polar side chains: selectivity/solubility
Palbociclib	Targeted therapy	CDK4/6 inhibitor	HR+/HER2− breast cancer	Kinase inhibitor	Piperazine: solubility; tertiary amines: bioavailability; pyridine N: CDK4 selectivity; cyclopentane: CDK6 hydrophobic position
Abemaciclib	Targeted therapy	CDK4/6 inhibitor	HR+/HER2− breast cancer	Kinase inhibitor	More lipophilic → better CNS penetration; NH H-bonding; propyl group: selectivity
Gomekli (mirdametinib)	Targeted therapy	MEK1/2 inhibitor	Neurofibromatosis type 1	Kinase inhibitor	H-bond donor: MEK1 hinge; halogen: potency
Dostarlimab-gxly	Immunotherapy	PD-1 inhibitor	dMMR (deficient mismatch repair) endometrial cancer	Checkpoint inhibitor	Biologic SAR: Fc-region amino acid substitutions
Retifanlimab	Immunotherapy	PD-1 inhibitor	Merkel cell carcinoma	Checkpoint inhibitor	IgG1 mAb with variable region mutations
Nivolumab + hyaluronidase-nvhy	Immunotherapy	Subcutaneous PD-1 inhibitor	Melanoma, NSCLC, others	Checkpoint inhibitor	Hyaluronidase: tissue permeability
Remestemcel-l-rknd (Ryoncil)	Immunotherapy	Mesenchymal stromal cell therapy	Pediatric steroid-refractory acute GVHD	Cell therapy	No SAR; activity *via* surface proteins (CD105, CD73)
Fam-trastuzumab deruxtecan	Antibody–drug conjugate	HER2-directed ADC (Topo I payload)	HER2-low/ultralow breast cancer	Antibody–drug conjugate	Linker stability, cleavable linker, exatecan payload
Datopotamab deruxtecan	Antibody–drug conjugate	TROP2-directed ADC	HR+/HER2− breast cancer	Antibody–drug conjugate	Linker/topoisomerase SAR drives selectivity and potency
Emrelis	Antibody–drug conjugate	c-Met-targeted ADC	NSCLC with high c-Met expression	Antibody–drug conjugate	Likely similar ADC SAR; structural info needed
Treosulfan	Cytotoxic chemotherapy	Alkylating agent	AML, MDS (transplant conditioning)	Alkylating agent	Diols improve water solubility
Sotorasib + panitumumab	Combination therapy	KRAS G12C inhibitor + EGFR blockade	KRAS G12C-mutant colorectal cancer	Combination therapy	Acrylamide: KRAS binding; lipophilic tail: cell permeability, oral bioavailability
Encorafenib + cetuximab + mFOLFOX6	Combination therapy	BRAF inhibitor + EGFR inhibitor + chemo	BRAF V600E-mutated colorectal cancer	Combination therapy	Pyrazole: π–π interaction; sulfonamide: BRAF pocket
Acalabrutinib + bendamustine + rituximab	Combination therapy	BTK inhibitor + chemo	Untreated mantle cell lymphoma	Combination therapy	Propynamide: BTK covalent binding; methyl group: ↓ ITK off-target
Avmapki Fakzynja	Other targeted therapy	KRAS-targeting combo (likely kinase inhibitor)	Low-grade serous ovarian cancer	Targeted therapy	Likely kinase scaffold; needs structural clarification
Avutometinib	Targeted therapy	MEK inhibitor	(Not clearly stated)	Kinase inhibitor (likely)	Pyrimidin-yloxy: MEK H-bonding; fluorinated ring: bioavailability; sulfamoylamino: solubility; chromenone methyl: dual inhibition
Defactinib	Targeted therapy	FAK inhibitor	(Not clearly stated)	Kinase inhibitor	Pyrimidine: H-bonding, planarity; *para*-fluorophenyl: hydrophobic pocket & stability; tertiary amine linker: alignment & PK optimization

***Structure–activity relationship***

**Fig. 2:**
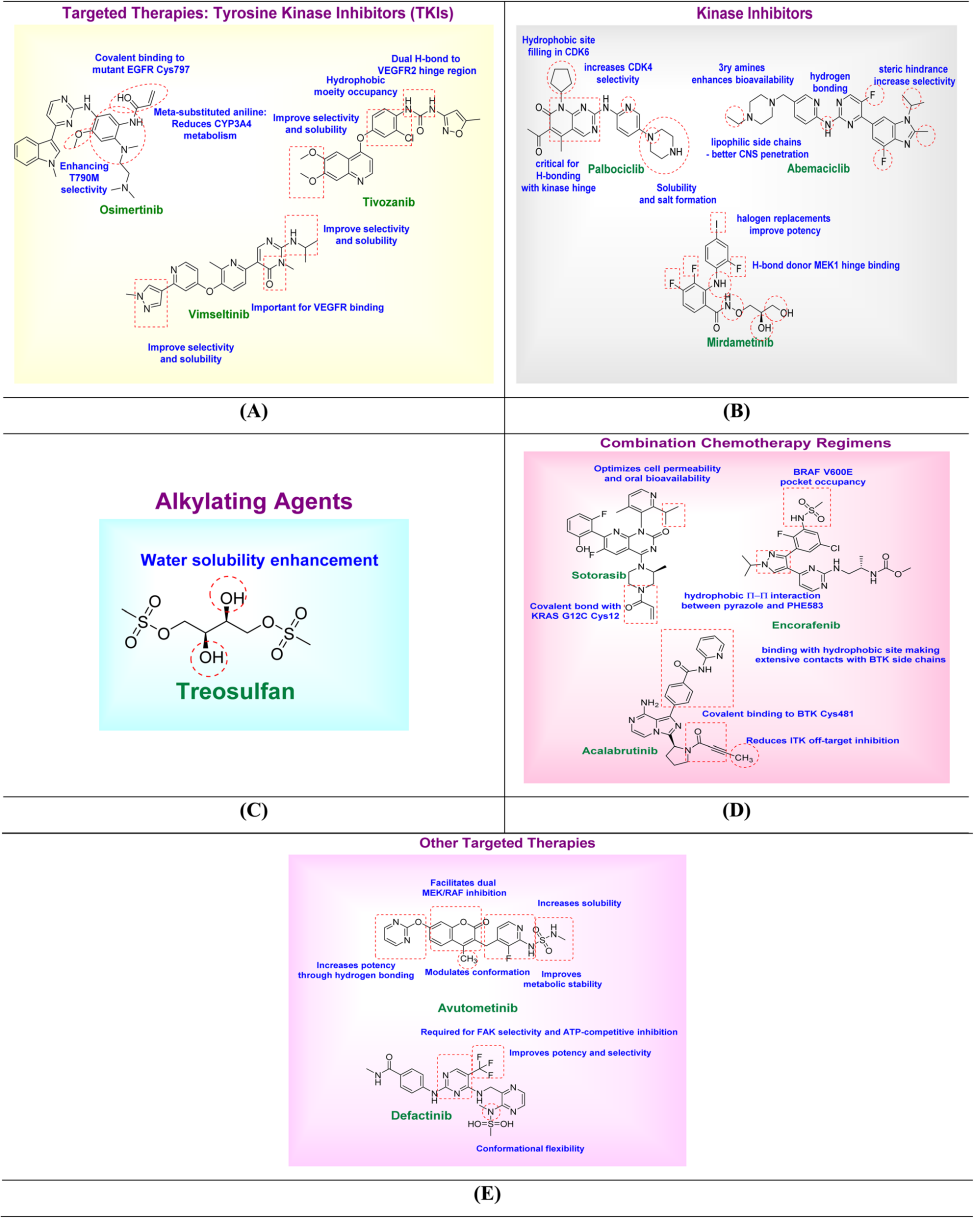
SAR illustration of some FDA-approved drugs: tyrosine kinase inhibitors (A), kinase inhibitors (B), alkylating agents (C), combination chemotherapy regimens (D), and other targeted therapies (E).

Osimertinib is designed to irreversibly target mutant EGFR harboring the T790M resistance mutation. The acrylamide warhead plays a pivotal role by covalently binding to the cysteine residue (Cys797) in the mutant EGFR kinase domain (SI Fig. S1A). A methoxy group positioned on the aromatic ring selectively blocks wild-type EGFR interaction, thereby improving mutant selectivity. Additionally, *meta*-substituted aniline reduces metabolic degradation by inhibiting CYP3A4-mediated oxidation, enhancing the stability of the drug and systemic exposure. Unlike first-generation reversible EGFR inhibitors (gefitinib, erlotinib) that fail against the T790M resistance mutation, and second-generation covalent inhibitors (afatinib, dacomitinib) that inhibit both mutant and wild-type EGFR, causing dose-limiting toxicities, the third-generation inhibitor AZD9291 (osimertinib) was rationally designed to selectively target T790M. It achieves this through an acrylamide warhead that covalently modifies Cys797 and peripheral substitutions that improve mutant selectivity and reduce metabolic clearance. This selective design confers potent T790M inhibition with markedly improved clinical tolerability. Also, the pyrrolo-pyridine scaffold demonstrated superior activity and selectivity compared to the indole analogue. Likewise, the acrylamide warhead proved more effective than the dimethylamino-butenamide counterpart. At the C4 position of the aniline ring, replacement of the *N*,*N*,*N′*-trimethylethylenediamine side chain with an *N*,*N*-dimethylamino-ethylenesulfoxide group produced a compound equipotent to osimertinib but with improved selectivity. In addition, substitution at the C5 position of the pyrimidine ring with chloro or bromo groups enhanced potency compared to the unsubstituted derivatives^80,81^ (Fig. 2A).

###### 2.1.1.1.2. Tivozanib (Fotivda)

Tivozanib is a potent and highly selective oral inhibitor of vascular endothelial growth factor receptor-1, -2, and -3 (VEGFR-1, -2, and -3), making it a promising anti-angiogenic agent for solid tumors, particularly renal cell carcinoma (RCC).^82^ By blocking VEGF-mediated signaling, tivozanib effectively reduces tumor vascularization and progression. Its unique pharmacological profile is characterized by high selectivity for VEGFR isoforms, which reduces off-target effects associated with other multi-kinase inhibitors.^83^ Tivozanib has a long elimination half-life, enabling once-daily oral dosing and improving patient compliance.^84^ The FDA approved tivozanib in 2021 for the treatment of adults with relapsed or refractory renal cell carcinoma (RCC) following at least two prior systemic therapies, based on data demonstrating favorable progression-free survival and tolerability^85^ (Table 1).

***Structure–activity relationship***

Tivozanib utilizes a quinoline-based scaffold. A urea linker enables dual hydrogen bonding with the VEGFR-2 hinge region, providing strong kinase binding. The incorporation of a chlorophenyl group facilitates hydrophobic interactions, improving binding affinity. Moreover, the presence of hydrogen bond acceptors and polar side chains supports selectivity among VEGFR isoforms and enhances water solubility for better pharmacokinetics. The presence of an electron-withdrawing group attached to the urea moiety enhanced both EGFR and VEGFR-2 inhibitory activities compared to analogues lacking such substitution. Introduction of an oxygen bridge favored VEGFR-2 inhibition, whereas replacement of the oxygen with sulfur shifted the activity profile toward stronger EGFR inhibition^86^ (Fig. 2A).

###### 2.1.1.1.3. Vimseltinib (Romvimza)

Vimseltinib, approved under the trade name romvimza in 2025, is a switch-control TKI that selectively targets colony-stimulating factor 1 receptor (CSF1R). CSF1R is overexpressed in macrophage-rich tumors such as tenosynovial giant cell tumor (TGCT), which plays a key role in the recruitment and proliferation of tumor-associated macrophages. By inhibiting CSF1R, vimseltinib disrupts the tumor microenvironment and induces regression of macrophage-driven tumors.^87^ The pharmacokinetic (PK) profile supports oral administration, though detailed PK parameters are not yet widely published. Clinical trials have demonstrated significant reductions in tumor size and symptomatic relief, leading to its FDA approval in patients with symptomatic, inoperable TGCT^88^ (Table 1).

***Structure–activity relationship***

Vimseltinib structure includes an amide linker known for hinge region binding and polar side chains that enhance both selectivity and solubility. These features ensure effective inhibition of CSF1R-driven signaling pathways implicated in tumor-associated macrophage activity. Replacement of the dimethylamino group in some analogues with a diethylamino group increased CSF1R potency and selectivity, whereas substitution of the 2-methyl group on the pyridine ring caused a marked reduction in CSF1R selectivity^89^ (Fig. 2A).

##### 2.1.1.2. Kinase inhibitors

###### 2.1.1.2.1. Palbociclib (Ibrance)

Palbociclib is an orally available, selective cyclin-dependent kinase (CDK) 4 and 6 inhibitor, introduced as a targeted therapy for hormone receptor-positive (HR+), human epidermal growth factor receptor-2 (HER2)-negative advanced breast cancer. CDK4/6 are critical regulators of cell cycle progression from G1 to S phase, and their inhibition by palbociclib results in hypophosphorylation of the retinoblastoma (Rb) protein, cell cycle arrest, and decreased tumor cell proliferation.^90^ Palbociclib has a favorable safety profile and is commonly used with endocrine therapy such as letrozole or fulvestrant. It is administered in 28-day cycles (21 days on, 7 days off), with dose adjustments based on neutropenia or other toxicities.^91^ Approved by the FDA in 2015, palbociclib significantly improves progression-free survival in HR+/HER2− patients, making it a mainstay of targeted breast cancer therapy. Gomekli (mirdametinib)–mitogen-activated protein kinases 1 and 2 (MEK1/2) inhibitor^92^ (Table 1).

***Structure–activity relationship***

Palbociclib is characterized by the presence of a piperazine ring, which significantly improves aqueous solubility and facilitates salt formation for formulation purposes. The tertiary amines within the piperazine also enhance oral bioavailability. A pyridine nitrogen linked to the piperazine ring is crucial for binding CDK4 selectively. Furthermore, a cyclopentane moiety contributes to a tight hydrophobic site filling, enhancing CDK6 inhibition. Substitution of the aniline side chain with a 2-aminopyridine moiety in some derivatives resulted in preferential inhibition of CD4 over CD2. Incorporation of a cyclopentyl group improved the physical properties compared to other analogues, while replacement of the acetyl group with bromine increased lipophilicity^93^ (Fig. 2B).

###### 2.1.1.2.2. Abemaciclib (Verzenio)

Abemaciclib is another oral CDK4/6 inhibitor approved for the treatment of HR+/HER2− advanced or metastatic breast cancer. Unlike palbociclib, abemaciclib allows for continuous daily dosing due to a different toxicity profile, particularly lower rates of neutropenia.^94^ It inhibits CDK4 more potently than CDK6, and this selectivity is thought to contribute to its efficacy and tolerability. Abemaciclib also demonstrates single-agent activity and is approved for use in both monotherapy and combination regimens.^95^ Its mechanism involves Rb-mediated G1 arrest, resulting in anti-proliferative effects in estrogen receptor-positive (ER+) breast cancer cells. Pharmacokinetically, it has a shorter half-life (∼18 hours) and is primarily metabolized by CYP3A. The FDA approved abemaciclib in 2017, and subsequent trials confirmed its role in adjuvant and metastatic settings^96^ (Table 1).

***Structure–activity relationship***

Structurally related to palbociclib, abemaciclib incorporates additional lipophilic side chains, which facilitate blood–brain barrier penetration—an advantage for treating central nervous system (CNS) metastases. The NH group contributes hydrogen bonding critical for kinase binding, while steric hindrance from the propyl moiety improves selectivity against off-target kinases. In the benzo[*d*]imidazole series, replacement of the *N*_1_-isopropyl group with smaller substituents such as methyl or ethyl, or with bulkier groups that introduce steric hindrance, resulted in decreased activity compared to the parent isopropyl analogue^93,97^ (Fig. 2B).

###### 2.1.1.2.3. Mirdametinib (Gomekli)

Mirdametinib is a highly selective MEK1/2 inhibitor developed for the treatment of neurofibromatosis type 1 (NF1)-associated plexiform neurofibromas.^98^ These tumors arise due to mutations in the NF1 gene, leading to hyperactivation of the Ras-Raf-MEK-ERK pathway. Mirdametinib blocks MEK phosphorylation and downstream ERK signaling, resulting in reduced tumor growth and symptom burden in NF1 patients.^99^ The drug is orally bioavailable and is suitable for use in both adult and pediatric populations. Approved by the FDA in 2025, mirdametinib represents a landmark therapy for patients with symptomatic, inoperable plexiform neurofibromas. Early-phase studies demonstrated durable tumor shrinkage and pain reduction, establishing MEK inhibition as a viable strategy in NF1 (ref. 100) (Table 1).

***Structure–activity relationship***

A key hydrogen bond donor in the mirdametinib structure enables binding to the MEK1 hinge region. Strategic halogen replacements on the aromatic ring enhance binding potency and improve pharmacodynamic effects, supporting efficacy in MEK-driven malignancies.^101^ Attachment of various nitrobenzoyl derivatives to the oxygen of the amide group resulted in decreased activity compared to the unsubstituted analogue^102,103^(Fig. 2B).

The future of targeted therapies in oncology is evolving across multiple drug classes. For TKIs, next-generation strategies focus on the development of allosteric inhibitors, covalent reversible TKIs, dual-target designs, and brain-penetrant scaffolds to overcome resistance and improve CNS penetration.^104^ In the case of CDK4/6 inhibitors, future directions emphasize the creation of CDK-selective degraders (PROTACs), expansion into CDK2/7/9 inhibitors, and combination strategies with endocrine therapy or immunotherapy to mitigate resistance and broaden therapeutic application.^105^ Meanwhile, for MEK/RAF pathway inhibitors, research is increasingly focused on pan-RAF and ERK inhibitors, synergistic combinations with KRAS inhibitors, and targeting adaptive feedback loops within MAPK signaling to prolong clinical benefit and prevent relapse.^106^

#### 2.1.2. Immunotherapies

##### 2.1.2.1. Checkpoint inhibitors (PD-1/PD-L1)

###### 2.1.2.1.1. Dostarlimab-gxly (Jemperli)

Dostarlimab-gxly is a humanized IgG4 monoclonal antibody that selectively binds to the programmed death-1 (PD-1) receptor expressed on activated T cells. By blocking PD-1 interaction with its ligands PD-L1 and PD-L2, dostarlimab restores T-cell-mediated immune responses against tumor cells, effectively enhancing anti-tumor immunity.^107^ It gained FDA approval in 2021 for the treatment of adult patients with mismatch repair-deficient (dMMR) recurrent or advanced endometrial cancer, a subset characterized by high mutational burden and responsiveness to immune checkpoint inhibition. Pharmacokinetically, dostarlimab has a half-life of approximately 25 days, allowing convenient dosing every 3 to 6 weeks.^107^ Clinical trials demonstrated durable response rates, with manageable immune-related adverse effects such as pneumonitis and colitis. Its mechanism exploits the concept of tumor immune evasion *via* checkpoint proteins, a paradigm-shifting approach that has transformed cancer therapeutics^108^ (Table 1).

###### 2.1.2.1.2. Retifanlimab (Zynyz)

Retifanlimab is a fully humanized IgG4 anti-PD-1 monoclonal antibody developed for immune checkpoint blockade. Approved in 2023, it is indicated for patients with metastatic or recurrent Merkel cell carcinoma (MCC), a highly aggressive neuroendocrine skin cancer often associated with Merkel cell polyomavirus.^107^ Retifanlimab disrupts PD-1-mediated immune suppression, thereby reinvigorating cytotoxic T-cell activity against tumor cells. Clinical trials showed a significant objective response rate in MCC patients, including durable remissions in virus-positive and virus-negative tumors.^109^ Pharmacodynamically, retifanlimab blocks PD-1 with high affinity, similar to other PD-1 inhibitors, and its safety profile is consistent with immune-related toxicities characteristic of checkpoint inhibitors^110^ (Table 1).

###### 2.1.2.1.3. Nivolumab and hyaluronidase-nvhy (Opdivo Qvantig)

Nivolumab is a well-established anti-PD-1 monoclonal antibody with broad indications across multiple solid tumors.^111^ The recent FDA approval (2024) of the subcutaneous formulation combines nivolumab with recombinant human hyaluronidase (rHuPH20), an enzyme that temporarily degrades hyaluronan in the extracellular matrix to enhance drug dispersion and absorption.^112^

This formulation maintains nivolumab's potent PD-1 blockade while offering improved patient convenience and reduced administration time compared to intravenous infusions. Pharmacokinetics remain comparable, with sustained plasma concentrations ensuring effective immune checkpoint inhibition. The combination aims to improve the quality of life without compromising efficacy or safety, a significant advance in immunotherapy delivery^113^ (Table 1).

These monoclonal antibodies were developed using rational antibody engineering. Strategies included variable region (VH/VL) modifications to fine-tune PD-1 binding affinity and Fc engineering to reduce effector functions such as ADCC. Dostarlimab was engineered as a humanized IgG4 antibody with durable receptor occupancy. Retifanlimab was optimized for high binding affinity and pharmacokinetic properties. Nivolumab was designed with an IgG4 backbone to prevent Fc receptor-mediated T-cell depletion.^114–116^

***Structure–activity relationship***

Dostarlimab, retifanlimab, and nivolumab are monoclonal antibodies targeting PD-1. Their SAR lies primarily in the engineered variable regions that modulate antigen binding affinity. Specific amino acid substitutions and glycosylation patterns in the Fc region are optimized to reduce effector function, thereby minimizing unwanted immune activation.^117^

##### 2.1.2.2. Cell therapy

###### 2.1.2.2.1. Remestemcel-l-rknd (Ryoncil)

Remestemcel-l is an allogeneic mesenchymal stromal cell (MSC) therapy derived from healthy donor bone marrow. Approved by the FDA in 2024 for steroid-refractory acute graft-*versus*-host disease (SR-aGVHD) in pediatric patients, this cell therapy exploits the immunomodulatory and anti-inflammatory properties of MSCs.^118^ The cells secrete bioactive molecules such as transforming growth factor-β (TGF-β), interleukin-10 (IL-10), and prostaglandin E2, which collectively suppress T-cell proliferation and shift the immune response toward tolerance.^119^ Additionally, MSCs promote tissue repair and inhibit fibrosis, which is crucial in the context of GVHD. Clinical trials have demonstrated improved survival and reduced inflammatory markers in treated children, positioning remestemcel-l as a valuable therapeutic option where corticosteroids fail^120^ (Table 1).

The synthesis strategy is based on *ex vivo* expansion of allogeneic mesenchymal stromal cells (MSCs) derived from healthy donors. These cells are engineered and optimized for immunomodulatory cytokine secretion (*e.g.*, IL-10, TGF-β). The therapeutic design relies on cellular secretome optimization and donor cell stability, with emphasis on manufacturing consistency, viability, and safety during large-scale expansion and cryopreservation.^121,122^

***Structure–activity relationship***

Although traditional SAR of remestemcel-l is not applicable, its pharmacological activity is driven by surface marker expression (*e.g.*, CD73, CD105) and secreted immunomodulatory factors that influence host immune responses.^123^

#### 2.1.3. Antibody–drug conjugates (ADCs)

##### 2.1.3.1. Fam-trastuzumab deruxtecan-nxki (Enhertu)

Fam-trastuzumab deruxtecan (DXd) is a next-generation ADC that targets HER2, a receptor overexpressed in breast cancer cells. The monoclonal antibody trastuzumab is linked *via* a cleavable peptide linker to a potent topoisomerase I inhibitor (DXd).^124^ Upon binding to HER2-positive cells, the ADC is internalized, and the cytotoxic payload is released intracellularly, inducing DNA damage and cell death. Enhertu's drug-to-antibody ratio (DAR) is high (∼8), improving payload delivery efficiency.^125^ Unlike earlier HER2-targeted therapies, it is effective in HER2-low expressing tumors, expanding treatment options. Clinical trials have demonstrated significant progression-free survival and overall response rates in patients with HER2-low metastatic breast cancer. Toxicity includes interstitial lung disease, which requires careful monitoring^126^ (Table 1).

Enhertu was synthesized by conjugating trastuzumab (anti-HER2) to the topoisomerase I inhibitor DXd *via* a cleavable tetrapeptide linker. Using maleimide-thiol or lysine coupling, the process achieves a high DAR (∼8). The linker ensures plasma stability and lysosomal release, allowing a bystander effect to kill HER2-low tumor cells.^127^

###### 2.1.3.1.1 Structure–activity relationship

This antibody–drug conjugate targets HER2 and includes a topoisomerase I inhibitor payload. The stability of the cleavable linker ensures tumor-selective release of the cytotoxic drug. The exatecan derivative used as a payload is potent and membrane-permeable, allowing for a bystander killing effect, which expands the therapeutic window, particularly in HER2-low tumors.^128^

##### 2.1.3.2. Datopotamab deruxtecan (Datroway)

Datopotamab deruxtecan is an ADC targeting trophoblast cell surface antigen 2 (TROP2), a transmembrane glycoprotein widely expressed in epithelial cancers, including breast cancer.^129^ The antibody is conjugated to a topoisomerase I inhibitor payload, which, after internalization, induces DNA damage leading to apoptosis. Approved in 2025 for hormone receptor-positive, HER2-negative breast cancer, it demonstrates efficacy in tumors with high TROP2 expression.^130^ The cleavable linker and membrane-permeable payload facilitate a bystander effect, killing adjacent tumor cells regardless of TROP2 expression heterogeneity. Clinical studies report favorable response rates with manageable hematologic and gastrointestinal side effects^131^ (Table 1).

###### 2.1.3.2.1 Structure–activity relationship

A TROP2-targeted ADC sharing the same payload and linker chemistry as trastuzumab deruxtecan. Structural modifications to the mAb enhance its hydrophilicity and stability, enabling a controlled release mechanism that reduces systemic toxicity while maintaining potent antitumor activity.^132^ Site-specific conjugation maintains a DAR ∼8, supporting selective intracellular release and strong bystander killing, even in TROP2-heterogeneous tumors.^133^

##### 2.1.3.3. Emrelis

Emrelis is a novel ADC targeting the c-Met receptor, a tyrosine kinase implicated in tumor proliferation, metastasis, and resistance in non-squamous non-small cell lung cancer (NSCLC).^134^ The antibody component binds c-Met with high affinity, enabling selective delivery of the cytotoxic payload to c-Met-overexpressing tumor cells. The payload induces apoptosis *via* DNA damage. Approved in 2025, Emrelis offers a targeted therapy option for patients with high c-Met expression, who traditionally have limited effective treatments. Pharmacologically, Emrelis represents an advancement in overcoming resistance mechanisms linked to c-Met signaling, with clinical trials confirming tumor regression and manageable adverse event profiles.^135^

###### 2.1.3.3.1 Structure–activity relationship

Though specific SAR data is lacking, Emrelis is presumed to follow similar principles of ADC design—selective antibody targeting, stable linker-payload conjugation, and release of a highly potent cytotoxic agent in tumor environments (Table 1).

The next wave of ADC development focuses on target diversification beyond HER2 and TROP2 to include receptors like HER3, c-Met, and B7–H3. Advances in linker chemistry—such as redox-sensitive or tumor pH-activated linkers—will improve payload release specificity. Additionally, the use of site-specific conjugation technologies (*e.g.*, enzymatic tags and engineered cysteine residues) will enable better control of DAR and reduce systemic toxicity. Novel payloads, including immune activators and transcriptional inhibitors, are being explored. Importantly, combination regimens with immune checkpoint inhibitors and kinase inhibitors are showing synergistic effects in early trials.^136^

#### 2.1.4. Cytotoxic chemotherapy

##### 2.1.4.1. Alkylating agents

###### 2.1.4.1.1. Treosulfan (Grafapex)

Treosulfan is a bifunctional alkylating agent that induces DNA cross-linking, leading to apoptosis. In January 2025, the FDA approved treosulfan in combination with fludarabine as a preparative regimen for allogeneic hematopoietic stem cell transplantation (alloHSCT) in patients aged 1 year and older with acute myeloid leukemia (AML) or myelodysplastic syndrome (MDS).^137^ This combination offers a reduced-toxicity conditioning regimen, expanding transplant eligibility to patients who may not tolerate traditional high-intensity regimens.^138^ Clinical studies demonstrated that treosulfan-based conditioning provided effective myeloablation with a favorable safety profile, making it a valuable option in the alloHSCT setting^139^ (Table 1).

Treosulfan is a prodrug that spontaneously converts *in vivo* to active bifunctional epoxides through non-enzymatic activation. Its synthesis involves sulfonation of diol precursors, enabling water solubility and improved myeloablative effects. The diol groups improve solubility and bioavailability over analogs like busulfan, making it favorable for reduced-toxicity stem cell transplant conditioning.^140^

***Structure–activity relationship***

Treosulfan is a water-soluble prodrug that undergoes non-enzymatic activation to produce bifunctional epoxide species. The inclusion of diol functional groups enhances its solubility, making it a useful agent for myeloablative conditioning with a reduced toxicity profile compared to busulfan. A wide range of stem cell toxicities was observed among the busulfan analogs, correlating with their ability to induce donor-type chimerism after bone marrow transplantation. *In vitro*, busulfan, PL63, PL26, PL108, and Treosulfan demonstrated comparable activity; however, *in vivo*, only busulfan and PL63 retained significant activity, which correlated with their bioavailability at the maximum tolerated dose^132,141^ (Fig. 2C).

#### 2.1.5. Combination chemotherapy regimens

##### 2.1.5.1. Sotorasib + panitumumab

Sotorasib is a covalent inhibitor targeting the kirsten rat sarcoma gene mutation, where the amino acid glycine (G) at position 12 is replaced by cysteine (C) (KRAS G12C) mutation, while panitumumab is an anti-EGFR monoclonal antibody. The combination was approved in early 2025 for the treatment of adult patients with KRAS G12C-mutated metastatic colorectal cancer (mCRC) who have received prior chemotherapy.^138^ This dual-targeted approach addresses the feedback activation of EGFR signaling that often limits the efficacy of KRAS inhibitors alone. Clinical trials demonstrated that the combination improved progression-free survival and objective response rates compared to historical controls^142–144^ (Table 1).

Sotorasib was synthesized *via* a modular approach, built around a pyrido[2,3-*d*] pyrimidine scaffold. The molecule contains an acrylamide warhead, which covalently binds to the KRAS G12C mutant *via* Cys12. A sulfonamide tail enhances binding and solubility, while a lipophilic side chain increases cell permeability and oral bioavailability.^145^

###### 2.1.5.1.1 Structure–activity relationship

Sotorasib employs an acrylamide group that covalently binds to Cys12 in the KRAS G12C mutant protein (SI Fig. S1B). The sulfonamide moiety enhances binding *via* hydrogen bonds in the switch-II pocket. A lipophilic tail contributes to improved cell permeability and oral absorption. ARS-1620, the first covalent KRAS G12C inhibitor, provided proof-of-concept but exhibited modest potency and suboptimal pharmacokinetics, limiting its clinical utility. Optimization of this scaffold led to sotorasib (AMG 510), which showed improved potency, enhanced S-II pocket engagement, and favorable pharmacokinetics, enabling successful clinical translation^146–148^ (Fig. 2D).

##### 2.1.5.2. Encorafenib + cetuximab + mFOLFOX6

Encorafenib is a B-Raf proto-oncogene, serine/threonine kinase, which is an inhibitor of a specific mutation where the amino acid valine (V) at position 600 is replaced by glutamic acid (E) (BRAF V600E) inhibitor, and cetuximab is an anti-EGFR antibody. When combined with the chemotherapy regimen mFOLFOX6 (comprising folinic acid, fluorouracil, and oxaliplatin).^149^ This triplet therapy was approved in 2024 for first-line treatment of BRAF V600E-mutant mCRC.^150^ The rationale for this combination stems from the need to inhibit multiple nodes in the MAPK pathway, which is hyperactivated in BRAF-mutant tumors. The randomized phase 3 study of encorafenib (enco) + cetuximab (cet) ± chemotherapy for first-line treatment (tx) of BRAF V600E-mutant (BRAFV600) metastatic colorectal cancer (mCRC) (BREAKWATER) trial reported a significant improvement in overall response rates and progression-free survival with this regimen^151^ (Table 1).

Encorafenib features a pyrazolopyrimidine scaffold with a sulfonamide substituent targeting the ATP-binding site of BRAF V600E. The dimethylpyrazole group avoids paradoxical MAPK activation. Synthesis involves coupling heteroaryl moieties and optimizing halogenated tails to adjust pharmacokinetics.^152^

###### 2.1.5.2.1 Structure–activity relationship

Encorafenib includes a dimethylpyrazole moiety that prevents paradoxical activation of the MAPK pathway, commonly seen with first-generation BRAF inhibitors. A sulfonamide group occupies the V600E-mutant BRAF ATP-binding site, while halogen substitutions fine-tune solubility and pharmacokinetics. The pyrazolo-pyrimidine scaffold directs the terminal phenyl/sulfonamide into the allosteric pocket, and replacing the pyrazole or pyrimidine, or removing the sulfonamide, generally reduces potency. Modifications of the sulfonamide *N*-substituent allowed tuning of potency, solubility, and safety, with modestly larger aryl or heteroaryl groups often maintaining or enhancing potency while affecting ADME properties^132,153^ (Fig. 2D).

##### 2.1.5.3. Acalabrutinib + bendamustine + rituximab

Acalabrutinib is a selective Bruton's tyrosine kinase (BTK) inhibitor.^154^ Its combination with bendamustine, an alkylating agent, and rituximab, an anti-CD20 monoclonal antibody, was approved in January 2025 for the treatment of previously untreated mantle cell lymphoma (MCL) in patients ineligible for autologous stem cell transplantation.^155^ This regimen leverages the synergistic effects of BTK inhibition and chemoimmunotherapy, resulting in improved progression-free survival and manageable toxicity profiles^156^ (Table 1).

Acalabrutinib was synthesized using a pyrazolopyrimidine core bearing a propynamide warhead for covalent binding to Cys481 of BTK. Its synthesis involves nucleophilic substitutions and coupling with the electrophilic warhead, with steric shielding groups minimizing off-target binding.^157^

###### 2.1.5.3.1 Structure–activity relationship

Acalabrutinib is a second-generation BTK inhibitor that features a covalent propynamide warhead, forming an irreversible bond with Cys481 (SI Fig. S1C). A pyrazolopyrimidine core provides hinge-binding specificity. The methyl substituent minimizes off-target activity, especially against Interleukin-2-inducible T-cell kinase (ITK), while polar groups increase aqueous solubility. Compared with ibrutinib, which irreversibly targets Cys481 but shows broad off-target kinase inhibition, acalabrutinib retains the covalent binding mechanism yet incorporates a refined hinge-binding headgroup that reduces off-target activity. This modification preserves potent BTK inhibition while improving tolerability, exemplifying how headgroup electronic and steric tuning can achieve greater selectivity^132,158,159^ (Fig. 2D).

The future of targeted therapies in oncology is advancing across multiple drug classes. For KRAS inhibitors, sotorasib has initiated a new era for KRAS G12C-mutant cancers; however, limitations due to resistance mutations and EGFR feedback have led to the development of pan-KRAS and G12D/G13D-selective inhibitors, with combinations involving EGFR antibodies (*e.g.*, panitumumab) and PD-1/PD-L1 inhibitors under active exploration to overcome adaptive resistance and enhance immunogenicity.^160^ For BRAF inhibitors, particularly encorafenib used with cetuximab and chemotherapy, future strategies include quadruplet regimens, adaptive dosing, and next-generation inhibitors to overcome resistance through alternate MAPK and PI3K-AKT pathways and avoid paradoxical activation, aiming for deeper and longer-lasting responses in BRAF V600E-mutant colorectal cancer. In the realm of BTK inhibitors, acalabrutinib is being refined through the development of non-covalent reversible inhibitors that retain efficacy against resistance mutations such as BTK C481S. Combined therapies with venetoclax and rituximab are improving remission depth, while emerging fixed-duration regimens aim to minimize chronic toxicity in mantle cell lymphoma and chronic lymphocytic leukemia.^161^

#### 2.1.6. Other targeted therapies

##### 2.1.6.1. Avmapki Fakzynja Co-pack (avutometinib + defactinib)

Avutometinib is a MEK1/2 inhibitor, and defactinib is a focal adhesion kinase (FAK) inhibitor. The combination was granted accelerated approval in May 2025 for the treatment of adult patients with KRAS-mutated recurrent low-grade serous ovarian cancer (LGSOC)^162,163^ who have received prior systemic therapy. This dual inhibition strategy targets both the MAPK pathway and the tumor microenvironment, addressing the intrinsic resistance mechanisms in LGSOC. Clinical studies demonstrated meaningful responses and disease control in this patient population^164^ (Table 1).

Avutometinib was synthesized around a chromen-2-one scaffold with a pyrimidin-2-yloxy group for MEK/RAF dual binding. Substituents such as fluoropyridine and sulfamoylamino groups enhance metabolic stability and solubility. Defactinib features a pyrimidine core, *para*-fluorophenyl ring, and tertiary amine linker, assembled through amide and aryl coupling.^165,166^

###### 2.1.6.1.1 Structure–activity relationship

Avutometinib is a dual MEK and RAF inhibitor with a chromen-2-one core. A pyrimidin-2-yloxy substituent improves MEK binding *via* hydrogen bonding. A fluorinated pyridine ring enhances metabolic stability and lipophilicity. Methylsulfamoylamino groups improve solubility, and the methyl group on the chromenone modulates its conformation for dual kinase engagement.^132^ Defactinib's pyrimidine core enables key hydrogen bonding and maintains planarity for effective binding. The *para*-fluorophenyl group fits into a hydrophobic site, improving affinity and metabolic stability. Its tertiary amine linker ensures proper alignment and enhances pharmacokinetic properties^167^ (Fig. 2E).

##### 2.1.6.2. Romvimza (vimseltinib)

Vimseltinib is an oral, selective inhibitor of the colony-stimulating factor 1 receptor (CSF1R), a tyrosine kinase implicated in the pathogenesis of tenosynovial giant cell tumor (TGCT).^168^ Approved in early 2025, vimseltinib offers a non-surgical treatment option for patients with symptomatic TGCT where surgery would result in significant morbidity. By inhibiting CSF1R, vimseltinib reduces the proliferation of tumor-associated macrophages, leading to tumor regression and symptom improvement^88^ (Table 1).

Vimseltinib is a selective CSF1R TKI with a tricyclic core scaffold and hinge-binding heterocycles. Its synthesis incorporates a urea or amide linker connecting to a polar head group for improved selectivity and oral bioavailability.^169^

##### 2.1.6.3. Mirdametinib (Gomekli)

Mirdametinib is an oral MEK1/2 inhibitor approved in February 2025 for the treatment of adults^170^ and pediatric patients aged 2 years and older with neurofibromatosis type 1 (NF1)^171^ who have symptomatic, inoperable plexiform neurofibromas. By inhibiting the MAPK pathway, mirdametinib reduces tumor growth and associated symptoms.^170^ Clinical trials demonstrated significant tumor shrinkage and improved quality of life in pediatric and adult patients^100^ (Table 1).

Mirdametinib is an MEK1/2 inhibitor containing a substituted indazole ring and amide linkage. It selectively blocks MEK activity in the MAPK pathway. Synthesis involves the coupling of indazole with urea or carbamate side chains to enhance stability and kinase selectivity.^101^

The future prospects: dual-pathway inhibition is gaining momentum in oncology, exemplified by the combination of avutometinib (MEK/RAF inhibitor) and defactinib (FAK inhibitor), which targets both tumor signaling and the microenvironment, showing promise in KRAS-mutated cancers like LGSOC.^172^ Future strategies include multi-functional single agents and combinatorial regimens extended to pancreatic and triple-negative breast cancers, with MEK/FAK plus immunotherapy also under evaluation. Meanwhile, CSF1R inhibitors such as vimseltinib are expanding from TGCT to immunosuppressive solid tumors like glioblastoma and liver cancer, often in synergy with checkpoint inhibitors to enhance T-cell infiltration and deplete tumor-associated macrophages, with additional applications in fibrosis and autoimmunity.^173^ Finally, MEK inhibitors like mirdametinib are being advanced for RASopathies and pediatric MAPK-driven tumors, with a focus on improving CNS penetration, tolerability, and integrating with liquid biopsy-based precision dosing and combination regimens involving BRAF or PI3K inhibitors.^101^

### 2.2. Investigational anticancer drugs in clinical trials

#### 2.2.1. Targeted small molecules

##### 2.2.1.1. Divarasib (GDC-6036)

Divarasib is a covalent, selective inhibitor of the KRAS G12C oncoprotein that binds the switch II pocket of KRAS in its inactive GDP-bound state. This prevents KRAS activation and inhibits downstream MAPK pathway signaling, leading to antiproliferative effects in tumors harboring the KRAS G12C mutation. In a multicenter Phase I/II trial, divarasib demonstrated a confirmed overall response rate (ORR) of 53% in KRAS G12C-mutant NSCLC and 29% in colorectal cancer (CRC), with median progression-free survival (PFS) of 13.1 months (NSCLC) and 5.6 months (CRC). Treatment-related adverse events (TRAEs) were mainly low-grade, with only 12% of patients experiencing grade 3/4 events and no treatment-related mortality^174^ (Table 2).

**Table 2:** Investigational anticancer drugs in clinical trials

Drug name	Drug class	Mechanism of action	Indication	Key clinical outcome/phase	Structure–Activity relationship (SAR)
Divarasib (GDC-6036)	KRAS inhibitor	Covalent inhibitor of KRAS G12C (switch II pocket)	NSCLC & CRC (KRAS G12C+)	ORR 53% (NSCLC); PFS 13.1 mo – Phase I/II	C7-substitution (*e.g.*, trifluoromethyl-pyridine) and warhead reactivity influence covalent binding to KRAS G12C
Glecirasib (JAB-21822)	KRAS inhibitor	Covalent KRAS G12C inhibitor	Solid tumors (KRAS G12C+)	ORR 48%, PFS 8.2 mo, OS ∼13.6 mo – Phase IIb	1,8-Naphthyridine scaffold optimized for solubility and metabolic stability; enhances irreversible KRAS G12C inhibition
PRT543	Epigenetic inhibitor	PRMT5 inhibitor (splicing and epigenetic regulation)	Ovarian cancer, MDS	CR in 1 ovarian case; responses in hematologic malignancies	PRMT5 inhibitors with π–π interactions at Phe327; basic amines in pharmacophore tuned for selectivity and stability
Tulmimetostat (CPI-0209)	Epigenetic inhibitor	Dual EZH1/EZH2 inhibitor (chromatin modulation)	ARID1A-mutant cancers	∼25% PRs; thrombocytopenia, GI AEs (gastrointestinal adverse events) – Phase I/II	EZH1/2 dual inhibition *via* chromatin interaction; SAR focuses on the methyltransferase domain and selective toxicity
Pegargiminase (ADI-PEG20)	Enzyme therapy	Depletes extracellular arginine in ASS1-deficient tumors	Melanoma, glioblastoma	SD 41–50%, PFS 3–5 mo, OS ∼8–11 mo – Phase I	PEGylated arginine deiminase improves stability and tumor targeting in ASS1-deficient cancers
^225^Ac-PSMA-R2	Radiopharmaceutical	Actinium-225-labeled PSMA-targeted alpha emitter	mCRPC (prostate cancer)	PSA ≥50% decline in ∼47%, low toxicity – Phase I/II	PSMA-targeting and alpha emitter tuning optimize selective radiation delivery with minimized hematologic toxicity
Scancell SCIB1	DNA vaccine	Plasmid vaccine encoding TRP2/gp100 melanoma antigens	Resected advanced melanoma	100% OS at cut-off; 5 relapses only – Phase I/II	Immunogenic TRP2/gp100 plasmid vaccine optimized for T-cell response and minimal off-target expression
Gridegalutamide	PROTAC	Androgen receptor degradation + antagonism	mCRPC	PSA declines; strong receptor degradation – Phase I	Linker rigidity and AR binding domain shape enhance proteasomal degradation and androgen signaling blockade
Opaganib (ABC294640)	SK2 inhibitor	Inhibits sphingosine kinase 2	Solid tumors	Some synergy with chemo – Phase II/III	Selective SK2 inhibition achieved by hydrophobic core tuning and solubility-driven modifications
Sapacitabine	Nucleoside analog	DNA synthesis inhibitor, induces SSBs in S-phase	AML, MDS (elderly/unfit patients)	ORR 30–45%, OS ∼30% at 1 year – Phase II	S-Phase selective analog design ensures DNA incorporation; substitutions influence chain termination and SSB induction
KappaMab (MDX-1097)	Monoclonal antibody	Targets kappa-light chain on MM cells	Relapsed/refractory multiple myeloma	ORR 83% with combo; OS benefit – Phase IIb	Targeting epitope binding and antibody engineering improves MM cell selectivity and immune-mediated killing
Glesatinib (MGCD-265)	TKI (type II MET)	Dual MET and SMO kinase inhibitor	MET-amplified NSCLC	ORR ∼15%, low efficacy – Phase II	Type II MET inhibition *via* DFG-out stabilization; scaffold substitutions failed to improve efficacy in MET-amplified tumors

###### 2.2.1.1.1 Structure–activity relationship

**Fig. 3:**
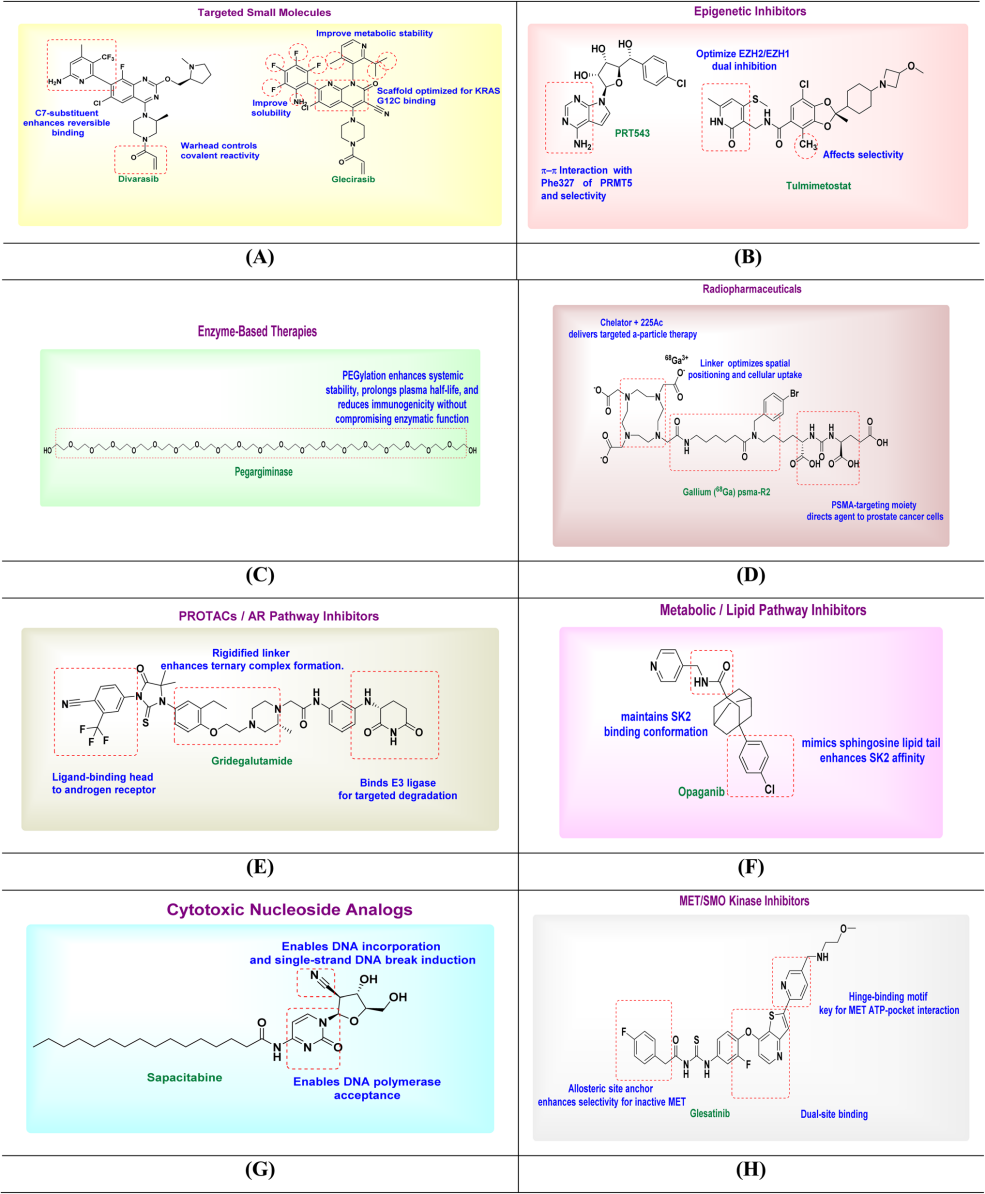
SAR illustration of investigational drugs: targeted small molecules (A), epigenetic inhibitors (B), enzyme-based therapies (C), radiopharmaceuticals (D), PROTACs/AR pathway inhibitors (E), metabolic/lipid pathway inhibitors (F), cytotoxic nucleoside analogs (G), and MET/SMO kinase inhibitors (H).

SAR studies of divarasib highlight the importance of the 2-amino-4-methyl-5-trifluoromethyl-pyridine moiety at C7, which enhances binding to the KRAS switch II pocket and properly orients the acrylamide warhead for covalent targeting of Cys12. Modifications in this region affect labeling kinetics, while C2-substitution is generally tolerated due to low steric hindrance. Divarasib also maintains a conserved binding pose across related guanosine triphosphateases (GTPases), supporting scaffold flexibility. These findings emphasize the need to optimize both reversible binding and warhead reactivity for selective KRAS(G12C) inhibition. Optimizing the covalent warhead geometry and electronics showed that subtle changes in position, electrophilicity, or leaving-group properties (*e.g.*, acrylamide *versus* fumarate-like warheads) could enhance the covalent reaction rate with Cys12 while maintaining selectivity. Structural studies indicated that precise positioning, rather than simply increasing electrophilicity, is key to improved activity^175,176^ (Fig. 3A).

##### 2.2.1.2. Glecirasib (JAB-21822)

Glecirasib is another irreversible KRAS G12C inhibitor in clinical development, selectively binding to and stabilizing the GDP-bound inactive form of KRAS. This blockade disrupts oncogenic signaling and tumor growth in KRAS G12C-mutated malignancies. In a Phase IIb trial involving 119 patients with advanced solid tumors, glecirasib achieved an objective response rate (ORR) of 48%, a disease control rate (DCR) of 86%, a median progression-free survival (PFS) of 8.2 months, and an overall survival (OS) of 13.6 months. Grade ≥3 TRAEs occurred in 38.7% of patients, though no treatment-related deaths were reported^177^ (Table 2).

###### 2.2.1.2.1 Structure–activity relationship

Glecirasib is a covalent KRAS^G12C inhibitor developed using structure-based design. Its 1,8-naphthyridine-3-carbonitrile scaffold anchors in the switch II pocket, while a precisely positioned warhead enables selective covalent binding to Cys12 (SI Fig. S1D). Further SAR refinements improved solubility, reduced metabolic liabilities, and enhanced pharmacokinetics, resulting in a potent and well-balanced clinical candidate for KRAS^G12C-mutant cancers. Glecirasib (JAB-21822) represents an optimized KRAS G12C inhibitor with nanomolar potency (IC_50_ ≈ 2.3 nM) and >500-fold selectivity over wild-type KRAS, contrasting with earlier covalent leads; notably, glecirasib also retains activity against secondary KRAS G12C resistance variants (*e.g.*, G12C/R68S, G12C/H95D/Q), highlighting how improved scaffold complementarity and physicochemical refinement translate into superior efficacy and resilience in KRAS G12C-targeted SAR^178,179^ (Fig. 3A).

#### 2.2.2. Epigenetic inhibitors

##### 2.2.2.1. PRT543

PRT543 is an orally available selective inhibitor of protein arginine methyltransferase 5 (PRMT5), an enzyme that regulates transcription and splicing *via* methylation of arginine residues on histones and RNA-binding proteins. Inhibition of PRMT5 results in altered gene expression, cell cycle arrest, and apoptosis in tumor cells. In early Phase I/II trials, PRT543 showed encouraging activity with complete remission in a case of ovarian cancer and additional responses in myeloid malignancies. Adverse effects included cytopenias, fatigue, and gastrointestinal symptoms^180^ (Table 2).

###### 2.2.2.1.1 Structure–activity relationship

PRT543 is a first-generation, SAM-competitive inhibitor of protein arginine methyltransferase 5 (PRMT5). It binds selectively to the S-adenosylmethionine (SAM) pocket, forming key hydrogen-bond and electrostatic interactions with critical residues such as Glu392, Leu319, Asp419, Tyr324, Met420, Pro314, and Lys393, which normally stabilize SAM in the PRMT5 active site (SI Fig. S1E). Building on early clinical inhibitors like GSK3326595, which exhibited limited clinical tolerability, PRT543 was optimized to achieve both enhanced potency and improved oral exposure. This combination enabled its progression into clinical trials with clear evidence of target modulation in patients, highlighting how fine-tuning potency alongside PK properties is essential for advancing PRMT5 inhibitors^181–183^ (Fig. 3B).

##### 2.2.2.2. Tulmimetostat (CPI-0209)

Tulmimetostat is a dual enhancer of zeste homolog 2 and enhancer of zeste homolog 1 (EZH2/EZH1) inhibitor that targets polycomb repressive complex 2 (PRC2), disrupting transcriptional silencing of tumor suppressor genes. This compound shows efficacy in epigenetically dysregulated cancers, such as AT-rich interactive domain-containing protein 1A (ARID1A)-mutant endometrial carcinoma and lymphomas. Phase I/II clinical trial results demonstrated partial responses in ∼25% of patients, with tolerable side effects, including thrombocytopenia and gastrointestinal disturbances^184^ (Table 2).

###### 2.2.2.2.1 Structure–activity relationship

Tulmimetostat (CPI-0209) is a next-generation dual EZH1/EZH2 inhibitor designed to target the SET domain and overcome resistance to EZH2-specific agents. Its central aromatic scaffold enables strong binding, while SAR-driven substitutions balance dual inhibition and minimize off-target effects. Peripheral modifications improve permeability, oral bioavailability, and pharmacokinetics. Tulmimetostat shows enhanced activity in tumors with ARID1A mutations or EZH2 gain-of-function, supporting its potential as a broad-spectrum anticancer agent. Dual EZH1/EZH2 inhibition is preferred to prevent EZH1 compensation, as modifications that selectively reduce EZH1 affinity can decrease efficacy in certain ARID1A contexts. Therefore, the design maintains activity against both isoforms^185,186^ (Fig. 3B).

#### 2.2.3. Enzyme-based therapies

##### 2.2.3.1. Pegargiminase (ADI-PEG20)

Pegargiminase is a pegylated arginine deiminase that depletes circulating arginine, a critical amino acid for the growth of argininosuccinate synthetase 1 (ASS1)-deficient tumors, including melanoma and glioblastoma. In Phase I studies, pegargiminase combined with platinum-based chemotherapy yielded stable disease in about 50% of patients, with median PFS ranging from 3 to 5 months and OS between 8 and 11 months. Toxicities were mild and included fatigue, nausea, and transaminase elevations (Table 2).

###### 2.2.3.1.1 Structure–activity relationship

Pegargiminase is a pegylated arginine deiminase engineered to treat ASS1-deficient tumors by depleting extracellular arginine and inducing selective tumor cell death. Its native catalytic site ensures high specificity for l-arginine, while PEGylation enhances pharmacokinetics by improving stability, extending half-life, and reducing immunogenicity. The SAR is shaped by protein engineering rather than small-molecule modifications, balancing enzymatic activity with systemic tolerability for effective, sustained arginine depletion. PEGylation greatly enhances the therapeutic profile of arginine-degrading enzymes compared to their native forms. In ADI-PEG20, PEG conjugation extends circulation time from hours to days and reduces immunogenicity, enabling sustained arginine depletion. Similarly, pegylated arginases such as BCT-100 achieve far greater stability and exposure than the unmodified enzyme^187–189^ (Fig. 3C).

#### 2.2.4. Radiopharmaceuticals

##### 2.2.4.1. ^225^Ac-PSMA-R2

^225^Ac-PSMA-R2 is an alpha-emitting radioligand composed of Actinium-225 linked to a PSMA-targeting ligand, enabling high-energy radiation delivery to PSMA-positive prostate cancer cells. In the Satisfaction Phase I/II trial for metastatic castration-resistant prostate cancer (mCRPC), 47% of patients experienced ≥50% PSA reductions. Hematologic toxicity, particularly anemia and thrombocytopenia, was the primary dose-limiting factor^190^ (Table 2).

###### 2.2.4.1.1 Structure–activity relationship

^225^Ac-PSMA-R2 is an alpha-emitting radiopharmaceutical targeting PSMA-expressing prostate cancer cells in mCRPC. Its SAR centers on three key elements (tripartite design): a urea-based PSMA ligand for high tumor affinity and reduced off-target uptake, a stable chelator (*e.g.*, DOTA) to securely bind actinium-225, and a tailored linker whose length and hydrophilicity affect biodistribution and clearance. These optimized features enable selective tumor targeting and effective DNA damage *via* alpha emissions, ensuring therapeutic efficacy with controlled toxicity.^191^ Modifying the linker's lipophilicity or steric bulk, such as through aromatic or aliphatic spacers, in other analogues allowed tuning of tumor-to-kidney uptake ratios^192,193^ (Fig. 3D).

#### 2.2.5. DNA vaccines/immunotherapies

##### 2.2.5.1. Scancell SCIB1

Scancell SCIB1 is a DNA plasmid-based cancer vaccine encoding tyrosinase-related protein 2 (TRP-2) and glycoprotein 100 (gp100) melanoma-associated antigens, designed with an IgG1 Fc domain to enhance immune uptake and antigen presentation. In Phase I/II trials in resected melanoma, SCIB1 achieved a 100% survival rate at interim analysis, with T-cell activation detected in most patients and only five relapses^194^ (Table 2).

Its design strategy includes epitope refinement for MHC-I binding, plasmid backbone optimization for expression, and helper domain insertion for CD4^+^ activation.^195^

###### 2.2.5.1.1 Structure–activity relationship

SCIB1 is a DNA vaccine encoding melanoma-associated antigens TRP2 and gp100 fused to a T-cell activation domain, aiming to enhance anti-tumor immunity in advanced melanoma. Its SAR focuses on optimized antigen selection for immunogenicity, epitope refinement to boost major histocompatibility complex class I (MHC-I) binding and CD8^+^ T-cell responses, and a plasmid backbone engineered for efficient expression and safety. Inclusion of a helper domain further stimulates CD4^+^ T-cell activation and immune memory. These structural optimizations support SCIB1's clinical efficacy and low relapse rates in early studies.^196^

#### 2.2.6. PROTACs/AR pathway inhibitors

##### 2.2.6.1. Gridegalutamide

Gridegalutamide is a novel androgen receptor (AR) degrader using proteolysis-targeting chimera (PROTAC) technology. By hijacking the E3 ubiquitin ligase complex, it degrades the AR protein while antagonizing residual signaling, offering a dual mechanism of action. In early Phase I trials for metastatic castration-resistant prostate cancer (mCRPC), the drug achieved PSA reductions and potent AR knockdown with a favorable pharmacokinetic profile^197^ (Table 2).

###### 2.2.6.1.1 Structure–activity relationship

Gridegalutamide is a PROTAC-based AR antagonist and degrader developed to overcome resistance in metastatic castration-resistant prostate cancer (mCRPC). A PROTAC combining: (1) a high-affinity androgen receptor (AR) ligand, (2) E3 ligase recruiter (VHL or CRBN), and (3) optimized linker. Its SAR is defined by three optimized components: an AR-binding ligand with high affinity for both wild-type and mutant receptors, an E3 ligase ligand targeting cereblon, or the von Hippel-Lindau tumor suppressor protein (CRBN or VHL) selected for efficient degradation, and a tailored linker whose length and polarity influence ternary complex formation and cellular activity. These structural refinements enable potent AR degradation and enhanced efficacy in treatment-resistant prostate cancer. Linker length and flexibility are key SAR parameters, with optimized variants enhancing ternary complex formation and androgenic receptor turnover. Additionally, the choice of E3 ligase ligand (*e.g.*, VHL *versus* CRBN recruiters) influences degradation kinetics and off-target effects^193,198,199^ (Fig. 3E).

#### 2.2.7. Metabolic/lipid pathway inhibitors

##### 2.2.7.1. Opaganib (ABC294640)

Opaganib is a selective inhibitor of sphingosine kinase 2 (SK2), an enzyme that modulates inflammatory and oncogenic sphingolipid signaling. This oral drug is being evaluated in Phase II/III studies across solid tumors and has shown synergy with chemotherapeutic agents in preclinical models. Full efficacy data are pending^200^ (Table 2).

###### 2.2.7.1.1 Structure–activity relationship

Opaganib (ABC294640) is a selective oral sphingosine kinase 2 (SK2) inhibitor that disrupts cancer-promoting S1P signaling. Its SAR centers on a lipophilic amide scaffold featuring a naphthyl group, which enhances SK2 selectivity and membrane permeability. Refinement of the amide linker and nearby electronic features optimized binding orientation, potency, and metabolic stability. These modifications produced a compound with strong SK2 inhibition, anti-proliferative and anti-inflammatory effects, and good pharmacokinetics, while minimizing off-target activity. The most effective strategy to enhance potency was rigidifying the lipophilic tail to better occupy the J-shaped pocket, for example, by introducing an internal aryl or cycloaliphatic ring. Empirical studies on SphK2 inhibitors confirmed that this modification improves both potency and selectivity^201–203^ (Fig. 3F).

#### 2.2.8. Cytotoxic nucleoside analogs

##### 2.2.8.1. Sapacitabine

Sapacitabine is an oral deoxycytidine analog that induces single-stranded DNA breaks, leading to replication arrest and cell death. It is being studied in elderly patients with acute myeloid leukemia (AML) or myelodysplastic syndrome (MDS) who are ineligible for intensive chemotherapy. In Phase II studies, the drug yielded ORRs between 30–45%, with a 1-year survival rate of ∼30%. Common toxicities included neutropenia and febrile neutropenia^204^ (Table 2).

###### 2.2.8.1.1 Structure–activity relationship

Sapacitabine is an oral nucleoside analog prodrug derived from 2′-deoxycytidine, designed to selectively disrupt DNA synthesis in cancer cells. Its structure–activity relationship (SAR) centers on the 2′-C-cyano group, which enhances enzymatic stability and promotes DNA incorporation. Once metabolized to CNDAC inside cells, it causes single-strand DNA breaks that convert to double-strand breaks during replication, inducing cell death in dividing cells. The prodrug design improves oral bioavailability and systemic exposure. Overall, sapacitabine's SAR reflects a strategic integration of stability, selective cytotoxicity, and pharmacokinetics, making it suitable for AML and MDS treatment in elderly or unfit patients. Prodrug strategies, such as N4 lipidation or phosphate prodrugs, effectively enhance oral exposure, with sapacitabine's palmitoyl amide serving as a validated example^205,206^ (Fig. 3G).

#### 2.2.9. Monoclonal antibodies

##### 2.2.9.1. KappaMab (MDX-1097)

KappaMab is a humanized monoclonal antibody targeting the kappa-light chain on malignant plasma cells. In Phase IIb trials for relapsed/refractory multiple myeloma, when combined with lenalidomide and dexamethasone, it achieved an ORR of 83% compared to 45% with standard therapy and significantly prolonged OS. No severe infusion reactions were reported^207^ (Table 2).

###### 2.2.9.1.1 Structure–activity relationship

KappaMab is a chimeric monoclonal antibody designed to selectively target membrane-bound κ-free light chains (κ-FLCs) on malignant plasma cells in κ-restricted multiple myeloma. Its structure–activity relationship (SAR) focuses on optimizing CDRs in the variable region to achieve high specificity for κ-FLCs while avoiding normal B cells. SAR studies have shown that refined binding kinetics reduce interaction with circulating soluble κ-light chains. Additionally, the Fc region is engineered to promote strong antibody-dependent cell-mediated cytotoxicity (ADCC) activity, supporting NK cell-mediated cytotoxicity. This balance of specificity, immune activation, and pharmacokinetics underlies its potential for treating relapsed or refractory κ-light chain multiple myeloma (MM).^208^

#### 2.2.10. Mesenchymal–epithelial transition factor/smoothened (MET/SMO) kinase inhibitors

##### 2.2.10.1. Glesatinib (MGCD-265)

Glesatinib is a type II small-molecule TKI that inhibits MET and SMO, both implicated in tumor progression and survival. It showed modest antitumor activity in NSCLC patients harboring MET exon 14 skipping mutations, with an ORR of ∼15%. The trial was terminated early due to insufficient efficacy. Adverse events included gastrointestinal and hepatic toxicity^209^ (Table 2).

###### 2.2.10.1.1 Structure–activity relationship

Glesatinib is a type II TKI that selectively targets the MET receptor in its inactive, highly conserved sequence found in the activation loop of protein kinases (DFG-out) conformation and also inhibits SMO in the Hedgehog pathway. Its structure–activity relationship (SAR) involves a hinge-binding moiety for ATP-pocket anchoring and a hydrophobic tail that occupies the MET gene's allosteric back pocket, enhancing selectivity (SI Fig. S1F). SAR studies focused on optimizing aromatic substitutions and side chains for potency, particularly against MET mutations, while polar group additions improved solubility and oral bioavailability. Despite its dual-target design and rational SAR-driven optimization, clinical efficacy in MET-amplified NSCLC was limited, resulting in discontinued development. However, early clinical studies revealed suboptimal pharmacokinetics and incomplete target inhibition, resulting in modest efficacy compared with newer, highly selective type I MET inhibitors such as capmatinib and tepotinib. This underscores the importance of both binding mode and exposure in achieving translational success^210,211^ (Fig. 3H).

Future directions in investigational anticancer therapies are embracing multi-target and personalized approaches. KRAS inhibitors like divarasib and glecirasib are expanding to pan-KRAS, non-covalent allosteric agents, and PROTAC degraders, with combinations involving SHP2, EGFR, and SOS1 showing promise for overcoming resistance. Epigenetic inhibitors such as PRT543 and tulmimetostat are evolving toward dual-targeting designs, aided by AI drug discovery and checkpoint blockade integration. Enzyme-based therapies like pegargiminase are being optimized for longer half-life and lower immunogenicity.^212^

### 2.3. Withdrawn or discontinued anticancer drugs

#### 2.3.1. Antibody–drug conjugates (ADCs)

##### 2.3.1.1. Gemtuzumab ozogamicin (Mylotarg)

Mylotarg is a CD33-directed antibody–drug conjugate (ADC) composed of a recombinant humanized IgG4 monoclonal antibody linked to the potent cytotoxic antibiotic calicheamicin. Designed for acute myeloid leukemia (AML), Mylotarg binds to CD33-expressing leukemic blasts, is internalized, and releases calicheamicin in the lysosomes, triggering double-stranded DNA breaks and apoptosis. Originally approved in 2000 under accelerated approval, the drug was voluntarily withdrawn in 2010 after the Phase III Southwest Oncology Group (SWOG S0106) trial demonstrated increased induction mortality (5.7% *vs.* 1.4%) and no survival benefit. Later studies with fractionated lower doses, particularly the Acute Leukemia French Association (ALFA)-0701 (ALFA-0701) trial, showed reduced toxicity and improved outcomes, leading to FDA reapproval in 2017. This case reflects the importance of dose optimization in ADC therapy.^213^ From a medicinal chemistry perspective, the early design employed an acid-labile hydrazone linker, which was later shown to be relatively unstable in plasma, causing premature payload release and systemic toxicity^214^ (Table 3).

**Table 3:** Withdrawn or discontinued anticancer drugs

Drug name	Drug class	Mechanism of action	Indication	Reason for withdrawal	Year	Structure–activity relationship (SAR)
Gemtuzumab ozogamicin	ADC	CD33-targeted ADC with calicheamicin (DNA damage)	Relapsed AML	Increased early mortality (SWOG S0106)	2010	Linker and antibody specificity are critical; calicheamicin potency demands precise delivery. Modifications to linker or conjugation chemistry influence off-target toxicity
Belantamab mafodotin	ADC	BCMA-targeted ADC with MMAF (tubulin inhibitor)	Multiple myeloma	Failed PFS (DREAMM-3); corneal toxicity	2022–23	MMAF linked *via* a non-cleavable linker; efficacy depends on internalization. BCMA affinity and linker stability drive both activity and ocular toxicity profile
Sacituzumab govitecan	ADC	TROP2-targeted ADC with SN-38 (Topo I inhibitor)	Urothelial carcinoma	Failed OS (TROPiCS-04); high toxicity	2024	SN-38 payload is potent; linker design affects systemic exposure. High hydrophobicity and rapid release contribute to the bystander effect and toxicity
Camidanlumab tesirine	ADC	CD25-targeted ADC with PBD dimer (DNA crosslinking)	Hodgkin lymphoma, AML	Early response, but halted due to toxicity	2024	PBD dimer requires precise targeting due to high DNA binding potency; antibody selectivity and linker chemistry are critical for therapeutic index
Mobocertinib (Exkivity)	TKI	Irreversible EGFR exon 20 insertion inhibitor	NSCLC (EGFR exon 20)	Failed PFS *vs.* chemo (EXCLAIM-2)	2024	Acrylamide warhead covalently binds mutant EGFR. Modifications to aniline or quinazoline rings impact selectivity and resistance profile
Infigratinib (Truseltiq)	TKI	FGFR1–3 inhibitor (selective)	FGFR2+ cholangiocarcinoma	No PFS benefit (PROOF301)	2024	Diaryl urea scaffold; piperazine side chain enhances FGFR2 binding. SAR focused on FGFR isoform selectivity, hinge binding, and solubility enhancements
Copanlisib (Aliqopa)	TKI	IV PI3Kα/δ inhibitor	Follicular lymphoma	Inadequate benefit (CHRONOS-4)	2024	Morpholine ring targets PI3K hinge. Isoform selectivity driven by heteroaromatic substitutions. IV formulation limited optimization for oral PK
Melphalan flufenamide	Alkylator	Peptide–drug conjugate releasing melphalan (DNA crosslinking)	Relapsed multiple myeloma	Increased mortality; failed OS (OCEAN)	2024	Lipophilic prodrug enhances tumor uptake. Amino acid linker controls melphalan release rate; SAR focused on peptide stability and tumor-selective cleavage
Marqibo	Cytotoxic	Liposomal vincristine formulation (mitotic inhibition)	Ph-ALL	Market withdrawal (limited clinical use)	2022	Liposomal encapsulation extends circulation and reduces neurotoxicity. Modifications at C3 and C4 often reduce tubulin binding. Substitution on the indole ring system can disrupt the conformational flexibility required for effective binding
Vibostolimab	Immunotherapy	Anti-TIGIT monoclonal antibody	Solid tumors	Development halted (lack of efficacy)	2024	Antibody epitope binding affects functional blocking. Engineering the Fc region affects immune activation and durability
Favezelimab	Immunotherapy	Anti-LAG-3 monoclonal antibody	Solid tumors	Poor efficacy in trials; program discontinued	2024	SAR is linked to epitope recognition. Affinity maturation and Fc optimization may enhance the response

###### 2.3.1.1.1 Structure–activity relationship

Gemtuzumab ozogamicin is an antibody–drug conjugate targeting CD33 on leukemic blasts in AML. Its SAR is defined by a high-affinity humanized IgG4 antibody, an acid-cleavable linker, and a calicheamicin payload. The linker ensures intracellular release within lysosomes while remaining stable in circulation. SAR studies on calicheamicin focused on structural modifications to retain potency while improving systemic tolerability.^215^ Overall, the design balances selective delivery, controlled release, and potent DNA damage for effective and targeted cytotoxicity.^216^

##### 2.3.1.2. Belantamab mafodotin (blenrep)

Blenrep is a first-in-class ADC targeting B-cell maturation antigen (BCMA), conjugated to monomethyl auristatin F (MMAF), a tubulin polymerization inhibitor. Approved in 2020 for heavily pretreated multiple myeloma, blenrep acts *via* targeted cytotoxicity and immune-mediated cell killing. However, the Phase III DRiving excellence in approaches to multiple myeloma (DREAMM-3) trial showed no significant improvement in progression-free survival compared to pomalidomide/dexamethasone (HR 1.03), and notable adverse effects such as keratopathy and thrombocytopenia raised safety concerns. Following FDA consultation, GlaxoSmithKline (GSK) withdrew the drug from the U.S. market in late 2022 and globally by March 2023, emphasizing the challenge of balancing efficacy with unique ADC toxicities.^217^ From a medicinal chemistry point of view, these toxicities were linked to the choice of payload and linker design, unlike calicheamicin-based ADCs, blenrep used MMAF (a charged auristatin derivative) attached *via* a non-cleavable linker, meaning the entire antibody–linker–drug complex accumulates inside the cell after lysosomal degradation. While this design reduces systemic free drug exposure compared to cleavable linkers, the intracellular retention of MMAF has been associated with off-target uptake in corneal epithelial cells, leading to ocular toxicity^218^ (Table 3).

###### 2.3.1.2.1 Structure–activity relationship

Belantamab mafodotin is an ADC targeting BCMA in multiple myeloma. Its SAR features an afucosylated anti-BCMA antibody to enhance ADCC, a non-cleavable mc linker, and the monomethyl auristatin F (MMAF) payload, a tubulin inhibitor with limited diffusion, improving tumor selectivity and reducing systemic toxicity. The non-cleavable linker ensures intracellular MMAF release only after lysosomal degradation. This design optimizes immune engagement, payload confinement, and safety in refractory myeloma.^219^

##### 2.3.1.3. Trodelvy (sacituzumab govitecan-hziy)

Trodelvy is a TROP-2-directed ADC delivering SN-38, the active metabolite of irinotecan, a topoisomerase I inhibitor. Approved for urothelial carcinoma in 2021, Trodelvy showed promise in pretreated patients due to its bystander effect and internalization-dependent cytotoxicity. However, the confirmatory study evaluating sacituzumab govitecan Trodelvy in patients with metastatic or locally advanced unresectable urothelial cancer that has progressed after platinum-based chemotherapy and checkpoint inhibitor therapy (TROPiCS-04) Phase III trial failed to meet its primary overall survival endpoint (OS: 10.3 *vs.* 9.0 months; HR 0.86, *p* = 0.087), with increased toxicity and treatment-related deaths. As a result, the urothelial indication was voluntarily withdrawn in November 2024, though its use in triple-negative breast cancer continues^220^ The hydrophilic linker ensured tumor-selective release and bystander effect, but toxicity and OS failure in urothelial cancer led to the withdrawal of that indication^221^ (Table 3).

###### 2.3.1.3.1 Structure–activity relationship

Trodelvy is an ADC targeting Trop-2, composed of a high-affinity IgG1 antibody, the cytotoxic SN-38 payload, and a cleavable CL2A linker. SAR optimization includes a high DAR (8) for efficient payload delivery, a hydrophilic linker enabling tumor-selective release and bystander killing, and antibody engineering to enhance Trop-2 binding while limiting off-target effects. This design supports potent, selective activity in Trop-2-expressing cancers like triple-negative breast cancer (TNBC).^222^

##### 2.3.1.4. Camidanlumab tesirine

Although not yet FDA-approved, it was withdrawn from development and is worth noting. It is an ADC composed of an anti-CD25 antibody linked to a pyrrolobenzodiazepine (PBD) dimer toxin, targeting CD25-expressing malignancies such as Hodgkin lymphoma and AML. Early-phase data showed >30% complete responses, but its further development was halted due to toxicity and unclear long-term efficacy^223^ (Table 3). The cleavable linker is designed for efficient intracellular release, but once released, the pyrrolo[2,1-*c*][1,4]benzodiazepine (PBD) payload lacks a therapeutic window due to its extreme DNA cross-linking potency. As seen with other PBD-containing ADCs, the inability to control bystander toxicity has limited clinical utility.^224^

###### 2.3.1.4.1 Structure–activity relationship

Camidanlumab tesirine is a CD25-targeting ADC composed of a fully human IgG1 antibody, a PBD dimer payload, and a protease-cleavable linker. SAR optimization focused on high-affinity binding and efficient internalization, while reducing off-target effects on normal CD25^+^ immune cells. The PBD toxin induces DNA cross-links with high potency and minimal detectability, and the linker ensures plasma stability with intracellular release. Despite strong activity, clinical use was limited by immune-related toxicity due to CD25 expression on regulatory T cells.^225^

#### 2.3.2. Kinase inhibitors

##### 2.3.2.1. Mobocertinib (Exkivity)

Mobocertinib is an oral, irreversible TKI targeting EGFR exon 20 insertion mutations, a subset of non-small-cell lung cancer (NSCLC) with poor response to standard EGFR TKIs. It binds covalently to the cysteine-797 residue in the EGFR active site, blocking the downstream mitogen-activated protein kinase pathway, and the phosphoinositide 3-kinase pathway, including PI3K (MAPK and PI3K) pathways. Mobocertinib was granted accelerated approval in 2021; however, the confirmatory clinical trial investigating the use of mobocertinib in previously untreated patients with locally advanced or metastatic EGFR exon 20 insertion mutation-positive non-small cell lung cancer (EXCLAIM-2) Phase III trial showed no improvement in PFS *versus* platinum-based chemotherapy (median 9.6 months in both arms, HR 1.04). Due to a lack of efficacy and modest safety concerns (*e.g.*, diarrhea, QTc interval corrected for heart rate on an electrocardiogram prolongation), Takeda voluntarily withdrew the drug in July 2024. It was designed for exon 20 insertions, but off-target EGFR inhibition and lack of PFS benefit led to withdrawal.^226^ This case underscores that scaffold optimization must balance potency against mutant isoforms with tolerability in normal tissue, a central challenge in designing next-generation TKIs^227^ (Table 3).

###### 2.3.2.1.1 Structure–activity relationship

**Fig. 4:**
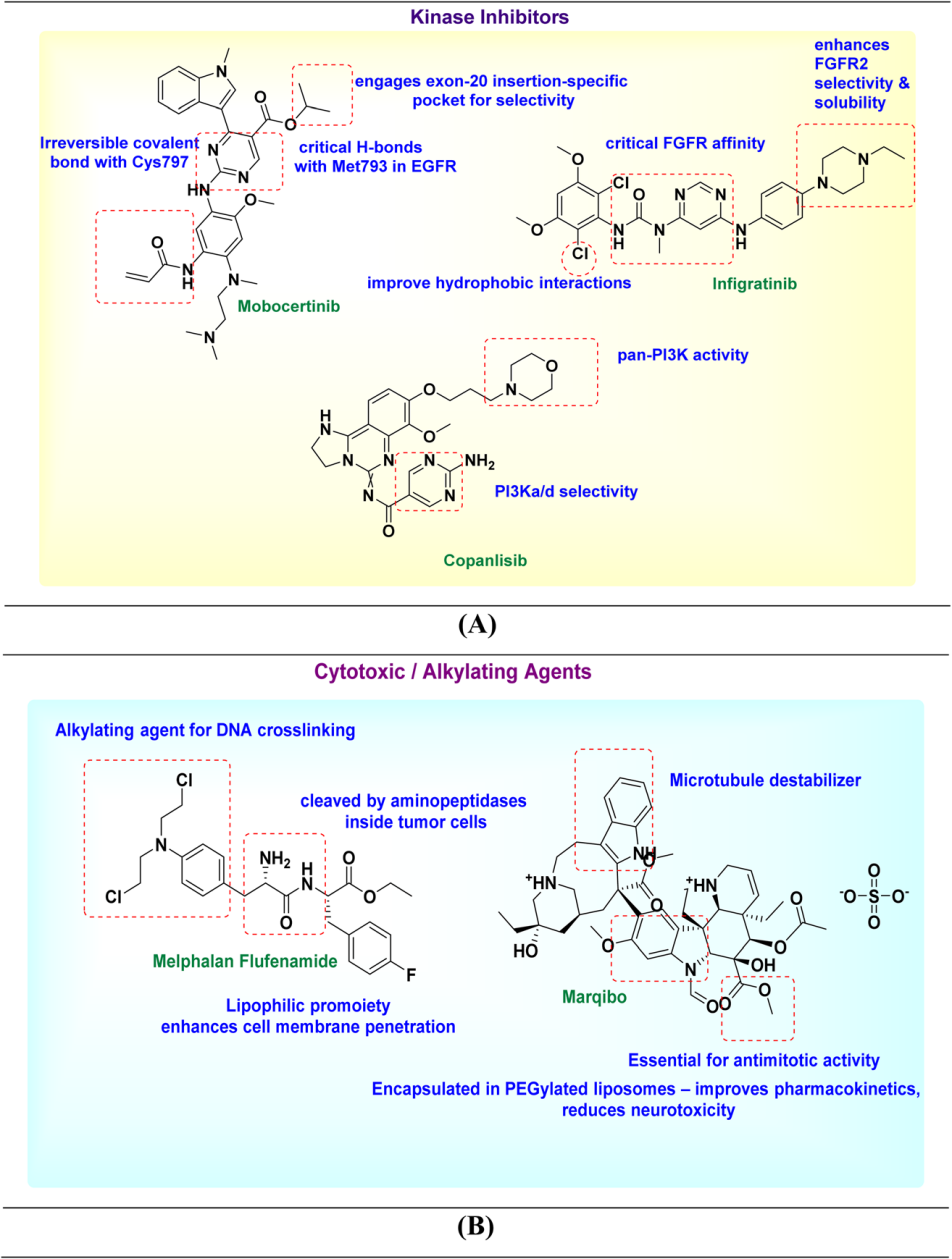
SAR illustration of withdrawn drugs: kinase inhibitors (A) and cytotoxic/alkylating agents (B).

Mobocertinib is an oral TKI designed to selectively inhibit EGFR exon 20 insertion mutations in NSCLC. Its SAR is centered on a pyrimidine core with C-4 aniline modifications that enhance mutant selectivity, and an acrylamide warhead that covalently binds Cys797 for irreversible inhibition (SI Fig. S1G). A methyl-piperazine moiety improves oral bioavailability and systemic exposure. SAR refinements in polarity and hydrogen bonding reduce wild-type EGFR inhibition, minimizing toxicity while maintaining potency. Unlike poziotinib, which exhibits higher potency but poor selectivity and unacceptable toxicity, mobocertinib achieves a balance of potency and tolerability, demonstrating how structure-guided optimization of R-group vectors enables exon-specific SAR despite clinical trade-offs^228,229^ (Fig. 4A).

##### 2.3.2.2. Infigratinib (Truseltiq)

Infigratinib is a selective FGFR1–3 inhibitor developed for FGFR2 fusion-positive cholangiocarcinoma. It acts by inhibiting FGFR-mediated oncogenic signaling, promoting apoptosis, and reducing angiogenesis. Initially approved based on single-arm trial results, its confirmatory trial focuses on infigratinib *versus* gemcitabine plus cisplatin in patients with advanced cholangiocarcinoma with an FGFR2 gene fusion/rearrangement (PROOF301) trial (*vs.* gemcitabine/cisplatin) failed to demonstrate progression-free survival benefit (PFS 7.4 *vs.* 8.0 months), leading to early trial termination and voluntary withdrawal in May 2024.^230^ The case of infigratinib illustrates the difficulty of designing highly selective FGFR inhibitors that maintain efficacy while avoiding class-related adverse effects such as hyperphosphatemia, nail toxicities, and ocular events^231,232^ (Table 3).

###### 2.3.2.2.1 Structure–activity relationship

Infigratinib is a selective oral FGFR1–3 inhibitor designed for FGFR-altered cancers, particularly FGFR2 fusion-positive cholangiocarcinoma. Its SAR is based on a pyrimidine–urea core that anchors the molecule in the FGFR ATP-binding site. Substitutions at C4 and C6 enhance hinge region binding, while a halogenated aryl group improves hydrophobic interactions. An ethylpiperazine moiety boosts solubility and oral bioavailability. SAR optimization focused on enhancing isoform selectivity and pharmacokinetics while minimizing off-target effects. A novel series of *N*-aryl-*N*′-pyrimidin-4-yl ureas was optimized through rational design of the aryl ring substitution pattern, yielding potent and selective inhibitors of FGFR1–3. Based on its favorable *in vitro* profile, compound 1 h (NVP-BGJ398) was selected for *in vivo* studies and demonstrated significant antitumor activity in RT112 bladder cancer xenografts overexpressing wild-type FGFR3, highlighting its potential as a therapeutic anticancer agent^233,234^ (Fig. 4A).

##### 2.3.2.3. Copanlisib (Aliqopa)

Copanlisib is a pan-class PI3K inhibitor, selectively inhibiting PI3K-α and PI3K-δ isoforms, with activity in B-cell malignancies such as follicular lymphoma. Administered intravenously, copanlisib was associated with adverse metabolic events (*e.g.*, hyperglycemia and hypertension) typical of PI3K-α inhibition. After its accelerated approval in 2017, the study investigating the efficacy and safety of copanlisib in combination with standard immunochemotherapy for relapsed indolent non-Hodgkin's lymphoma (CHRONOS-4) trial failed to confirm its clinical benefit, leading to voluntary market withdrawal in March 2024. Copanlisib highlights the trade-offs between oral bioavailability *versus* tolerability in PI3K inhibitors; its case reinforces the importance of developing next-generation isoform-selective or allosteric PI3K inhibitors that can minimize class-wide safety liabilities while preserving efficacy^235^ (Table 3).

###### 2.3.2.3.1 Structure–activity relationship

Copanlisib is an IV-administered pan-class I PI3K inhibitor with selectivity for PI3K-α and PI3K-δ. Its SAR centers on a morpholino-triazine core, with C4 and C6 substitutions optimized to enhance isoform selectivity while reducing PI3K-β/γ off-target effects. Fluorinated aryl groups improve metabolic stability and half-life. Time-dependent inhibition of PI3K-δ supports durable activity in B-cell malignancies and contributes to its clinical efficacy and tolerability. The 2,3-dihydroimidazo[1,2-*c*]quinazoline scaffold of copanlisib adopts a flat conformation in the p110 catalytic pocket and extends into a deeper-affinity site, unlike idelalisib, whose quinazoline occupies a selectivity pocket. Copanlisib shows higher potency across all class I PI3K isoforms, with tenfold preference for p110α/δ, leading to cell-type-specific cytotoxicity. Intermittent intravenous dosing reduces gastrointestinal toxicity compared with continuous oral idelalisib, and clinically, copanlisib demonstrates superior efficacy and tolerability in relapsed follicular lymphoma and activated B-cell DLBCL^236,237^ (Fig. 4A).

#### 2.3.3. Cytotoxic/alkylating agents

##### 2.3.3.1. Melphalan flufenamide (Pepaxto)

Melphalan flufenamide is a lipophilic peptide–drug conjugate of the alkylating agent melphalan, designed for enhanced tumor targeting *via* hydrolysis by aminopeptidase N. Upon intracellular release, melphalan induces DNA crosslinking, triggering cell death. The Phase III The optimal anti-coagulation for enhanced-risk patients post-catheter ablation for atrial fibrillation (OCEAN) trial (*vs.* pomalidomide/dexamethasone) failed to show an overall survival advantage and, concerningly, indicated higher mortality in the treatment arm (OS 19.8 *vs.* 25.0 months; HR 1.10). The FDA withdrew approval in February 2024, reflecting the limited utility of cytotoxic alkylators despite modern targeting approaches. It illustrates both the promise and pitfalls of peptide–drug conjugates. Although the prodrug strategy successfully improved delivery and enzyme-targeted activation, the disconnect between progression-free and overall survival outcomes emphasizes that rational design at the chemistry level must ultimately be validated by long-term clinical benefit^238^ (Table 3).

###### 2.3.3.1.1 Structure–activity relationship

Melphalan flufenamide is a peptide–drug conjugate that improves melphalan delivery *via* enzymatic activation in tumors. Its SAR features a lipophilic dipeptide linked to melphalan, enhancing membrane permeability and allowing intracellular release by aminopeptidases like CD13. Peptide substitution at the melphalan amine boosts tumor selectivity, while the flufenamide moiety and linker design influence permeability, release kinetics, and systemic stability, improving therapeutic index and reducing toxicity. Melphalan flufenamide delivers higher intracellular melphalan levels than free melphalan, yielding lower IC_50_ values and inducing apoptosis even in resistant multiple myeloma cells. Its activity depends on aminopeptidase *N*–mediated conversion and involves caspase activation, ROS generation, mitochondrial dysfunction, and DNA damage. It also inhibits cell migration and angiogenesis, shows superior tumor suppression in xenografts *versus* melphalan, and acts synergistically with lenalidomide, bortezomib, or dexamethasone^239,240^ (Fig. 4B).

##### 2.3.3.2. Marqibo (liposomal vincristine)

Marqibo was a reformulated liposomal form of vincristine for Ph-negative Acute Lymphoblastic Leukemia (ALL). It was quietly withdrawn from the market in May 2022 due to commercial and clinical limitations, involving neurotoxicity, which includes minimal differentiation from conventional vincristine, and logistical constraints of liposomal administration^241^ (Table 3).

###### 2.3.3.2.1 Structure–activity relationship

Marqibo is a liposomal formulation of vincristine that enhances efficacy and safety through formulation-based SAR. Encapsulation in sphingomyelin/cholesterol liposomes prolongs circulation, improves tumor accumulation *via* the EPR effect, and reduces neurotoxicity by limiting free drug exposure. While vincristine's structure remains unchanged, the delivery system optimizes pharmacokinetics and therapeutic index, highlighting the role of formulation in SAR. Encapsulation of vincristine in sphingomyelin/cholesterol liposomes (Marqibo) alters its pharmacokinetics and delivery, extending plasma half-life, increasing tumor accumulation through enhanced extravasation, and enabling sustained intratumoral release. These modifications allow higher cumulative dosing with improved tolerability and therapeutic index compared with non-liposomal vincristine^242,243^ (Fig. 4B).

#### 2.3.4. Immunotherapies

##### 2.3.4.1. Vibostolimab

Vibostolimab is a monoclonal antibody targeting T cell Immunoglobulin and ITIM domain (TIGIT), a co-inhibitory receptor on T cells and NK cells. It was hypothesized to enhance anti-tumor immune activity when combined with PD-1/PD-L1 blockade. However, Phase I/II studies failed to show meaningful additive efficacy, and development was discontinued in 2024^244^ (Table 3).

###### 2.3.4.1.1 Structure–activity relationship

Vibostolimab is a human IgG1 monoclonal antibody targeting TIGIT to restore T-cell and NK cell function in tumors. Its SAR emphasizes high-affinity binding to TIGIT's extracellular domain to block CD155 interaction without disrupting CD226 co-stimulation. Unlike Fc-silent anti-TIGIT antibodies, vibostolimab retains wild-type IgG1 Fc to enable ADCC and potential Treg depletion. Epitope targeting and preserved effector function were key SAR features supporting synergistic activity with PD-1 inhibitors in solid tumors.^245^

##### 2.3.4.2. Favezelimab

Favezelimab is a lymphocyte activation gene 3 (anti-LAG-3) monoclonal antibody that blocks LAG-3 signaling on exhausted T cells, promoting immune activation. Like vibostolimab, it failed to produce consistent responses in early trials and was terminated as a development program in the same year. These discontinuations reflect the ongoing challenge of translating checkpoint blockade beyond PD-1 and CTLA-4 (ref. 246) (Table 3).

###### 2.3.4.2.1 Structure–activity relationship

Favezelimab is an Fc-silent IgG4 monoclonal antibody targeting LAG-3 to restore T-cell function in tumors. Its SAR centers on high-affinity binding to the D1 domain of LAG-3, blocking MHC–II interaction without triggering ADCC. Fc mutations (*e.g.*, S228P) reduce immune effector functions, preserving beneficial T cells. SAR studies support its synergy with PD-1 inhibitors, with selective activity in LAG-3-high tumors, enabling effective dual checkpoint blockade with minimized immune toxicity.^247^

Future perspectives for withdrawn or discontinued anticancer drugs emphasize refining design strategies across different therapeutic classes. For ADCs such as Mylotarg, Blenrep, Trodelvy, and Camidanlumab, the future lies in creating more stable, tumor-specific linkers, site-specific conjugation for consistent DAR, and next-generation payloads with improved therapeutic windows, while bispecific ADCs and immune-modulating payloads are also advancing.^248^ For kinase inhibitors like Mobocertinib, Infigratinib, and Copanlisib, the focus will be on enhancing mutant isoform selectivity, reducing wild-type toxicity, and employing strategies like covalent reversible or allosteric inhibition, with combination therapies (*e.g.*, PROTACs, ADCs, or immunotherapies) being promising.^249^ In the case of discontinued cytotoxics such as Melphalan, Flufenamide, and Marqibo, future directions involve rational prodrug strategies, liposomal formulations, and enzyme-activated prodrugs, but these require predictive biomarkers to guide patient selection. Lastly, for immunotherapies like vibostolimab and favezelimab, which failed to achieve meaningful responses, the next wave will likely involve multi-checkpoint blockade (*e.g.*, TIGIT + PD-1 + LAG-3), Fc engineering for more precise immune modulation, and integration with cell therapies and vaccines to overcome resistance and improve durability of response.^250^

## 3. Medicinal chemistry insights driving modern drug development (Novelty)

### 3.1. Advances in linker design for antibody–drug conjugates (ADCs)

The chemical design of ADC linkers has matured from early, heterogeneous conjugation chemistries to highly engineered, site-specific approaches that materially improve stability, therapeutic index, and manufacturability.^251^ Early-generation maleimide cysteine conjugation produced heterogeneous drug-antibody ratios and risked premature payload release; in contrast, site-specific strategies (engineered cysteines, enzymatic methods, and small-molecule chemistries) yield more homogeneous conjugates with better pharmacokinetics and safety profiles.^252^ Modern linker selection is purpose-driven: cleavable linkers (protease-, pH-, or glutathione-sensitive) are chosen when payload release in the tumour microenvironment or lysosome is desired, whereas non-cleavable linkers are used to maximize plasma stability and alter the catabolite profile.^253^ Recent medicinal-chemistry advances have introduced stimuli-responsive and hydrophobicity-masking designs (photo-responsive, redox/iron-sensitive, and enzyme-triggered linkers) and refined site-specific conjugation to reduce off-target toxicity and improve therapeutic windows.^254^

### 3.2. Scaffold hopping in next-generation kinase inhibitors

Scaffold hopping, which is replacing the central core scaffold while preserving the pharmacophore that engages the target, is a mature, evidence-based tactic used to escape intellectual-property constraints, mitigate ADME/toxicity liabilities, and recover activity against resistant mutants.^255^ In the kinase field this strategy has enabled transition from early ATP-mimetic cores to chemically distinct hinge-binding heterocycles and rigidified scaffolds that preserve key interactions yet improve selectivity, metabolic stability, and brain penetrance where required.^256^ Practical examples across tyrosine-kinase programs show how scaffold replacement can retain target engagement while opening new vectors for optimization of solubility, metabolic soft-spots, and CNS exposure outcomes documented in both conceptual reviews and concrete kinase lead series.^257^

### 3.3. PROTAC chemistry *versus* classical SAR optimization

PROTACs represent a mechanistic and medicinal-chemistry paradigm shift.^258^ Classical SAR optimizes a single binding interaction (affinity/selectivity) to block function; PROTACs are heterobifunctional molecules that drive the formation of a productive ternary complex between the target protein and an E3 ligase to elicit ubiquitination and proteasomal degradation.^259^ Consequently, linker design, rather than only improving target affinity, becomes central: linker length, attachment geometry, rigidity/flexibility, and polarity strongly influence ternary-complex cooperativity, cellular permeability, and degradation kinetics.^260^ Recent advances (macrocyclization to pre-organize degrader geometry, photo-switchable PROTACs for spatiotemporal control, and in-cell click-assembled PROTACs/CLIPTAC approaches for modular assembly) illustrate distinct medicinal-chemistry levers that are not used in classical SAR workflows.^261,262^ These principles and reported examples are well documented in the PROTAC literature and mechanistic reviews.^263,264^

## 4. Conclusion

The past decade has witnessed unprecedented progress in anticancer drug development, with medicinal chemistry playing a central role in translating biological discoveries into clinically effective therapies. This review underscores how the intricate relationship between drug structure, molecular targets, and clinical efficacy has led to a paradigm shift—from conventional cytotoxic agents to precisely engineered molecules that exploit specific vulnerabilities in cancer cells. Medicinal chemistry has enabled the rational design of small-molecule inhibitors, monoclonal antibodies, ADCs, and emerging platforms such as PROTACs and molecular glues. These modalities have revolutionized the treatment landscape across a variety of malignancies, including solid tumors and hematologic cancers. Key milestones include the development of selective kinase inhibitors like osimertinib and palbociclib, immune checkpoint inhibitors such as dostarlimab, and ADCs like fam-trastuzumab deruxtecan that combine targeting precision with cytotoxic potency. Structural optimization based on SAR analysis has been pivotal in enhancing drug specificity, reducing off-target toxicity, and improving pharmacokinetic profiles.

Despite these advances, several challenges remain. Drug resistance, tumor heterogeneity, and immune evasion continue to limit long-term treatment efficacy. Moreover, the withdrawal of several anticancer agents due to suboptimal efficacy or unacceptable toxicity highlights the need for robust clinical validation and post-marketing surveillance. The failures of drugs like mobocertinib and belantamab mafodotin serve as critical reminders of the complexity of cancer biology and the importance of translational research. Future directions in anticancer drug discovery will rely heavily on interdisciplinary integration. Artificial intelligence and machine learning are beginning to reshape the early phases of drug design by predicting molecular interactions, optimizing lead compounds, and forecasting ADME/Tox profiles. Advances in epigenetic modulation, tumor microenvironment targeting, radiotheranostics, and cell-based therapies offer new horizons for therapeutic intervention. Additionally, drug delivery systems such as nanocarriers and liposomes, informed by chemical engineering principles, will further improve therapeutic index and tissue selectivity.

In summary, medicinal chemistry remains the engine driving the evolution of oncology therapeutics. As our understanding of cancer biology deepens and new technologies emerge, the synergistic interplay between molecular design, pharmacological innovation, and clinical translation will be crucial in achieving the ultimate goal of personalized, durable, and curative cancer treatment. This review highlights not only past and present achievements but also illuminates the promising future of anticancer drug development through the lens of medicinal chemistry.

## 5. Expert opinion

The past decade has marked a transformative period in anticancer drug development, with medicinal chemistry serving as the keystone in linking biological insights to therapeutic applications. The growing convergence of structural biology, pharmacology, and clinical oncology has enabled not just the synthesis of new drug candidates but also the realization of truly rational, mechanism-driven therapies. From kinase inhibitors and ADCs to PROTACs and radiotheranostics, the integration of SAR data into every stage of drug development is now standard. This trend is significantly impacting real-world outcomes by advancing treatment specificity, minimizing adverse effects, and improving cost-effectiveness through more durable responses.

The clinical implications of these innovations are substantial. Structure-guided design and SAR-based optimization have led to drugs with superior pharmacokinetic and safety profiles—osimertinib's brain-penetrant EGFR inhibition, or fam-trastuzumab deruxtecan's improved linker chemistry and payload delivery are just a few examples. In addition, medicinal chemistry's role in reformulating legacy drugs (*e.g.*, vincristine into liposomal Marqibo) or repurposing old scaffolds with better selectivity has made previously difficult targets viable. These advances could reshape treatment guidelines, particularly by expanding access to personalized oncology for malignancies with actionable mutations.

However, despite these successes, the translation of medicinal chemistry breakthroughs into routine clinical practice remains uneven. Cost, manufacturing complexity, and biomarker availability remain key barriers. Moreover, the accelerated approval of certain agents—followed by market withdrawal due to safety concerns or failed confirmatory trials—points to a need for improved translational validation. The cases of belantamab mafodotin and mobocertinib reflect how structural optimization alone is insufficient if real-world efficacy and tolerability are not adequately assessed across diverse patient populations.

Critical areas for improvement include enhancing the predictability of preclinical models and adopting more integrative pharmacological assessments that reflect tumor microenvironment complexities. Technical limitations also persist—namely, in the accurate modeling of protein–protein interactions, drug metabolism prediction, and resistance emergence. AI-driven drug design and molecular dynamics simulations offer promise, but widespread use is still hindered by data standardization issues and a lack of regulatory frameworks that support such novel methodologies.

Further research into emerging modalities such as epigenetic therapies, molecular glues, and immune-cell engagers will open new therapeutic windows. The interplay between medicinal chemistry and these domains will be crucial, as understanding the SAR of non-traditional small molecules (*e.g.*, E3 ligase recruiters in PROTACs) will demand more sophisticated design strategies. Although a definitive endpoint for this field is unlikely, given the dynamic nature of cancer evolution, the goal remains to achieve increasingly specific, durable, and safe responses.

While the field of oncology continues to diversify, the principles of medicinal chemistry will remain central to its progress. The future may indeed see immunotherapy, gene editing, and mRNA platforms dominate headlines, but these too will rely on structure-based chemistry to refine delivery, stability, and selectivity. Therefore, rather than competing disciplines, these technologies will likely converge under a unified medicinal chemistry framework.

By 2030, we envision a drug discovery ecosystem in which SAR modeling is driven by integrated AI platforms, seamlessly connecting omics data, patient-derived models, and structure-based predictions. Clinicians will have access to decision-support tools that predict drug efficacy and resistance mechanisms in real time, allowing personalized therapy selection not just based on genotype but on predicted ligand–target interactions. Drug development timelines will be shortened through iterative virtual screening cycles and continuous learning systems. Most importantly, the standard oncology care paradigm will shift toward ultra-targeted, SAR-informed therapeutic regimens designed to outpace resistance and maximize patient benefit.

## Author contributions

Conceptualization and supervision: Ahmed A. Al-Karmalawy; data collection, data curation, visualization, methodology, and writing – review & editing: Ahmed A. Al-Karmalawy, Mohamed E. Eissa, Nada A. Ashour, Tarek A. Yousef, Arwa Omar Al Khatib, Samia S. Hawas. All authors approved the submitted version of the manuscript.

## Conflicts of interest

The authors declared no conflict of interest.

## Supplementary Material

RA-015-D5RA05472A-s001

## Data Availability

The data supporting this article have been included as part of the SI. Supplementary information is available. See DOI: https://doi.org/10.1039/d5ra05472a.

## References

[cit1] Ottaiano A., Ianniello M., Santorsola M., Ruggiero R., Sirica R., Sabbatino F., Perri F., Cascella M., Di Marzo M., Berretta M., Caraglia M., Nasti G., Savarese G. (2023). Biology.

[cit2] Alqaraleha M., Kasabrib V., Muhanac F., Al-Najjarc B. O., Khleifatd K. M. (2024). J. Med. Pharm. Chem. Res..

[cit3] Basak D., Arrighi S., Darwiche Y., Deb S. (2021). Life.

[cit4] Al Taher R. S., Abbas M. A., Halahleh K., Sughayer M. A. (2024). Technol. Cancer Res. Treat..

[cit5] Koper K., Wileński S., Koper A. (2023). Phys. Sci. Rev..

[cit6] Belal A., Abdel Gawad N. M., Mehany A. B. M., Abourehab M. A. S., Elkady H., Al-Karmalawy A. A., Ismael A. S. (2022). J. Enzyme Inhib. Med. Chem..

[cit7] Cvrat M. and Sanat D. J. C., Chemotherapy Furthers Cancer by Killing T-Cells, 2021

[cit8] Munir M., Cheema A. Y., Ogedegbe O. J., Aslam M. F., Kim S. J. C. (2024). Cureus.

[cit9] El-Naggar A. M., Hassan A. M. A., Elkaeed E. B., Alesawy M. S., Al-Karmalawy A. A. (2022). Bioorg. Chem..

[cit10] Zeidan M. A., Ashour H. F., Yassen A. S. A., Abo Elmaaty A., Farag A. B., Sharaky M., Abdullah Alzahrani A. Y., Mughram M. H. A. L., Al-Karmalawy A. A. (2025). RSC Med. Chem..

[cit11] Herrera-Juárez M., Serrano-Gómez C., Bote-de-Cabo H., Paz-Ares L. (2023). Cancer.

[cit12] Barras B. J., Ling T., Rivas F. (2024). Molecules.

[cit13] Hammouda M. M., Elmaaty A. A., Nafie M. S., Abdel-Motaal M., Mohamed N. S., Tantawy M. A., Belal A., Alnajjar R., Eldehna W. M., Al-Karmalawy A. A. (2022). Bioorg. Chem..

[cit14] Ostios-Garcia L., Pérez D. M., Castelo B., Herradón N. H., Zamora P., Feliu J., Espinosa E. (2024). Cancer Metastasis Rev..

[cit15] Jiao Z., Wang G., Feng Z., Yan Z., Zhang J., Li G., Wang Q., Feng D. (2022). Front. Oncol..

[cit16] Abdel-Motaal M., Aldakhili D. A., Farag A. B., Elmaaty A. A., Sharaky M., Mohamed N. A., Shaaban S., Alzahrani A. Y. A., Al-Karmalawy A. A. (2024). RSC Med. Chem..

[cit17] Koller P. B., Othman T., Yang D., Mokhtari S., Samara Y., Blackmon A., Agrawal V., Pourhassan H., Ball B. J., Amanam I., Arslan S., Otoukesh S., Sandhu K. S., Aldoss I., Ali H., Salhotra A., Aribi A., Artz A., Becker P. S., Pullarkat V., Stewart F. M., Smith E. P., Stein A., Marcucci G., Forman S. J., Nakamura R., Al Malki M. M. (2025). Bone Marrow Transplant..

[cit18] Hammoud M. M., Nageeb A. S., Morsi M. A., Gomaa E. A., Elmaaty A. A., Al-Karmalawy A. A. (2022). New J. Chem..

[cit19] Alzahrani S. M., Al Doghaither H. A., Al-Ghafari A. B., Pushparaj P. N. (2023). Oncol. Rep..

[cit20] Jang J. Y., Kim D., Kim N. D. (2023). Int. J. Mol. Sci..

[cit21] Al-Karmalawy A. A., Elmaaty A. A., Magdy G., Radwan A. S., Alnajjar R., Shaldam M. A., Al Khatib A. O., Almujri S. S., Abdullah Alzahrani A. Y., Tawfik H. O. (2025). RSC Adv..

[cit22] Gaber A. A., Abo Elmaaty A., Sharaky M., Mosa A. A., Yahya Abdullah Alzahrani A., Shaaban S., Eldehna W. M., Al-Karmalawy A. A. (2024). Bioorg. Chem..

[cit23] Wordeman L., Vicente J. J. (2021). Cancers.

[cit24] Basak D., Arrighi S., Darwiche Y., Deb S. (2021). Life.

[cit25] Min H.-Y., Lee H.-Y. (2022). Exp. Mol. Med..

[cit26] Al-Karmalawy A. A., Nafie M. S., Shaldam M. A., Elmaaty A. A., Antar S. A., El-Hamaky A. A., Saleh M. A., Elkamhawy A., Tawfik H. O. (2023). J. Med. Chem..

[cit27] Abbass E. M., Al-Karmalawy A. A., Sharaky M., Khattab M., Alzahrani A. Y. A., Hassaballah A. I. (2024). Bioorg. Chem..

[cit28] El-Gamil D. S., Zaky M. Y., Maximous P. M., Sharaky M., El-Dessouki A. M., Riad N. M., Shaaban S., Abdel-Halim M., Al-Karmalawy A. A. (2024). Drug Dev. Res..

[cit29] Abourehab M. A. S., Alqahtani A. M., Youssif B. G. M., Gouda A. M. (2021). Molecules.

[cit30] Al-Karmalawy A. A., El-Subbagh H. I., Logoyda L., Lesyk R. B., El-Gamal M. I. (2023). Front. Chem..

[cit31] Wesley C. D., Sansonetti A., Neutel C. H. G., Krüger D. N., De Meyer G. R. Y., Martinet W., Guns P. J. (2024). Biology.

[cit32] El-Kalyoubi S., Elbaramawi S. S., Eissa A. G., Al-Ageeli E., Hobani Y. H., El-Sharkawy A. A., Mohamed H. T., Al-Karmalawy A. A., Abulkhair H. S. (2023). Future Med. Chem..

[cit33] Acharjee S. , Chauhan S., Pal R. and Tomar R. S., in Progress in Molecular Biology and Translational Science, ed. V. Singh and I. Mani, Academic Press, 2023, vol. 197, pp. 51–92 10.1016/bs.pmbts.2023.01.001 37019597

[cit34] Patnaik E., Madu C., Lu Y. (2023). Int. J. Mol. Sci..

[cit35] Zhong G., Chang X., Xie W., Zhou X. (2024). Signal Transduct. Targeted Ther..

[cit36] Song Y., Qing-Qing D., Yi-Ke N., Xiao-Ling X., Chao-Xiang C., Chen W. (2024). Int. J. Nanomed..

[cit37] Dewey J. A., Delalande C., Azizi S.-A., Lu V., Antonopoulos D., Babnigg G. (2023). J. Med. Chem..

[cit38] Yu X., Li D., Kottur J., Kim H. S., Herring L. E., Yu Y., Xie L., Hu X., Chen X., Cai L., Liu J., Aggarwal A. K., Wang G. G., Jin J. (2023). J. Med. Chem..

[cit39] Hong J., Li K., He J., Liang M. (2024). Bioconjugate Chem..

[cit40] Dean A. Q., Shen L., Twomey J. D., Zhang B. (2021). mAbs.

[cit41] Tarantino P., Ricciuti B., Pradhan S. M., Tolaney S. M. (2023). Nat. Rev. Clin. Oncol..

[cit42] Raavi, Koehler A. N., Vegas A. J. (2025). Chem. Rev..

[cit43] Li S., Pan W., Tao C., Hu Z., Cheng B., Chen J., Peng X. (2025). J. Med. Chem..

[cit44] Wu K., Kwon S. H., Zhou X., Fuller C., Wang X., Vadgama J., Wu Y. (2024). Int. J. Mol. Sci..

[cit45] Todorov L., Kostova I. (2023). Molecules.

[cit46] Kostova I. (2024). Inorganics.

[cit47] Abdolmaleki S., Aliabadi A., Khaksar S. (2024). Coord. Chem. Rev..

[cit48] Gao J., Karp J. M., Langer R., Joshi N. (2023). Chem. Mater..

[cit49] Zakaria M. Y., Elmaaty A. A., El-Shesheny R., Alnajjar R., Kutkat O., Ben Moussa S., Abdullah Alzahrani A. Y., El-Zahaby S. A., Al-Karmalawy A. A. (2024). RSC Adv..

[cit50] Mahmoud D. B., Ismail W. M., Moatasim Y., Kutkat O., ElMeshad A. N., Ezzat S. M., El Deeb K. S., El-Fishawy A. M., Gomaa M. R., Kandeil A., Al-karmalawy A. A., Ali M. A., Mostafa A. (2021). J. Drug Delivery Sci. Technol..

[cit51] Joy R. D., George J., John F. J. C. (2022). ChemistrySelect.

[cit52] Tafish A. M., El-Sherbiny M., Al-Karmalawy A. A., Soliman O. A. E., Saleh N. M. (2023). Int. J. Nanomed..

[cit53] El-Kalyoubi S., Khalifa M. M., Abo-Elfadl M. T., El-Sayed A. A., Elkamhawy A., Lee K., Al-Karmalawy A.
A. (2023). J. Enzyme Inhib. Med. Chem..

[cit54] Zhou W., Jia Y., Liu Y., Chen Y., Zhao P. (2022). Pharmaceutics.

[cit55] Lou J., Duan H., Qin Q., Teng Z., Gan F., Zhou X., Zhou X. (2023). Pharmaceutics.

[cit56] Pfister T. D., Reinhold W. C., Agama K., Gupta S., Khin S. A., Kinders R. J., Parchment R. E., Tomaszewski J. E., Doroshow J. H., Pommier Y. (2009). Mol. Cancer Therapeut..

[cit57] Bhatia N., Hazra S., Thareja S. (2023). Eur. J. Med. Chem..

[cit58] El-Masry T. A., El-Nagar M. M., Oriquat G. A., Alotaibi B. S., Saad H. M., El Zahaby E. I., Ibrahim H. A. (2024). Biomed. Pharmacother..

[cit59] Zhuang R., Xie R., Peng S., Zhou Q., Lin W., Ou Y., Chen B., Su T., Li Z., Huang H., Li K., Duan Y. (2025). J. Transl. Med..

[cit60] Scott A. M., Zeglis B. M., Lapi S. E., Scott P. J. H., Windhorst A. D., Abdel-Wahab M., Giammarile F., Paez D., Jalilian A., Knoll P., Korde A., Vichare S., Ayati N., Lee S. T., Lyashchenko S. K., Zhang J., Urbain J. L., Lewis J. S. (2024). Lancet Oncol..

[cit61] Holik H. A., Ibrahim F. M., Elaine A. A., Putra B. D., Achmad A., Kartamihardja A. H. S. (2022). Molecules.

[cit62] Kumar A., Singh A. K., Singh H., Vijayan V., Kumar D., Naik J., Thareja S., Yadav J. P., Pathak P., Grishina M., Verma A., Khalilullah H., Jaremko M., Emwas A.-H., Kumar P. (2023). Pharmaceuticals.

[cit63] Roesch F., Martin M. (2023). J. Radioanal. Nucl. Chem..

[cit64] Strosberg J., El-Haddad G., Wolin E., Hendifar A., Yao J., Chasen B., Mittra E., Kunz P. L., Kulke M. H., Jacene H. (2017). N. Engl. J. Med..

[cit65] Kratochwil C., Giesel F. L., Stefanova M., Benešová M., Bronzel M., Afshar-Oromieh A., Mier W., Eder M., Kopka K., Haberkorn U. (2016). J. Nucl. Med..

[cit66] Vallabhajosula S. , in Molecular Imaging and Targeted Therapy: Radiopharmaceuticals and Clinical Applications, Springer, 2023, pp. 1–19

[cit67] Jackson J. A., Hungnes I. N., Ma M. T., Rivas C. (2020). Bioconjug. Chem..

[cit68] Sreedevi B., Kishore N., Ghosh S., Kaur G. (2024). Int. J. Trends OncoScience.

[cit69] Salgueiro M. J. and Zubillaga M., Theranostic Nanoplatforms in Nuclear Medicine: Current Advances, Emerging Trends, and Perspectives for Personalized Oncology, Preprints, 2025, preprint, 10.20944/preprints202507.1404.v1

[cit70] Serghini A. , Portelli S. and Ascher D. B., in Computational Drug Discovery and Design, Springer, 2023, pp. 269–294

[cit71] Chakraborty S. , Chakraborty S., Sarkar B., Ghosh R., Roy S., Singh N. K. and Rakshit G., Scaffold hopping and de novo drug design, Computational Methods for Rational Drug Design, 2025, pp. 195–219

[cit72] Staszak M., Staszak K., Wieszczycka K., Bajek A., Roszkowski K., Tylkowski B. (2022). WIREs Comput. Mol. Sci..

[cit73] Zhavoronkov A., Ivanenkov Y. A., Aliper A., Veselov M. S., Aladinskiy V. A., Aladinskaya A. V., Terentiev V. A., Polykovskiy D. A., Kuznetsov M. D., Asadulaev A., Volkov Y., Zholus A., Shayakhmetov R. R., Zhebrak A., Minaeva L. I., Zagribelnyy B. A., Lee L. H., Soll R., Madge D., Xing L., Guo T., Aspuru-Guzik A. (2019). Nat. Biotechnol..

[cit74] (a) Monarch R. M. , Human-in-the-Loop Machine Learning: Active Learning and Annotation for Human-Centered AI, Simon and Schuster, 2021

[cit75] Azadinejad H., Farhadi Rad M., Shariftabrizi A., Rahmim A., Abdollahi H. J. D. (2025). Diagnostics.

[cit76] Ashiwaju B. I., Orikpete O. F., Uzougbo C. G. (2023). Matrix Sci. Pharma.

[cit77] Cross D. A., Ashton S. E., Ghiorghiu S., Eberlein C., Nebhan C. A., Spitzler P. J., Orme J. P., Finlay M. R., Ward R. A., Mellor M. J., Hughes G., Rahi A., Jacobs V. N., Red Brewer M., Ichihara E., Sun J., Jin H., Ballard P., Al-Kadhimi K., Rowlinson R., Klinowska T., Richmond G. H., Cantarini M., Kim D. W., Ranson M. R., Pao W. (2014). Cancer Discov..

[cit78] Mok T. S., Wu Y.-L., Ahn M.-J., Garassino M. C., Kim H. R., Ramalingam S. S., Shepherd F. A., He Y., Akamatsu H., Theelen W. S. M. E. (2017). N. Engl. J. Med..

[cit79] FDA , FDA D.I.S.C.O.: Osimertinib for Non-Small Cell Lung Cancer, https://www.fda.gov/drugs/resources-information-approved-drugs/fda-approves-osimertinib-chemotherapy-egfr-mutated-non-small-cell-lung-cancer

[cit80] Finlay M. R., Anderton M., Ashton S., Ballard P., Bethel P. A., Box M. R., Bradbury R. H., Brown S. J., Butterworth S., Campbell A., Chorley C., Colclough N., Cross D. A., Currie G. S., Grist M., Hassall L., Hill G. B., James D., James M., Kemmitt P., Klinowska T., Lamont G., Lamont S. G., Martin N., McFarland H. L., Mellor M. J., Orme J. P., Perkins D., Perkins P., Richmond G., Smith P., Ward R. A., Waring M. J., Whittaker D., Wells S., Wrigley G. L. (2014). J. Med. Chem..

[cit81] Patil B. R., Bhadane K. V., Ahmad I., Agrawal Y. J., Shimpi A. A., Dhangar M. S., Patel H. M. (2024). Bioorg. Med. Chem..

[cit82] Rini B. I., Pal S. K., Escudier B. J., Atkins M. B., Hutson T. E., Porta C., Verzoni E., Needle M. N., McDermott D. F. (2020). Lancet Oncol..

[cit83] Meza L., McDermott D. F., Escudier B., Hutson T. E., Porta C., Verzoni E., Atkins M. B., Kasturi V., Pal S. K., Rini B. (2023). Oncologist.

[cit84] Sakellakis M., Zakopoulou R. (2023). Cureus.

[cit85] FDA , FDA approves tivozanib for relapsed or refractory advanced renal cell carcinoma, https://www.fda.gov/drugs/resources-information-approved-drugs/fda-approves-tivozanib-relapsed-or-refractory-advanced-renal-cell-carcinoma

[cit86] Yousefbeyk F., Ghasemi S. J. P. S. (2024). Pharm. Sci..

[cit87] Lee A. (2025). Vimseltinib: First Approval: A. Lee. Drugs.

[cit88] FDA , FDA approves vimseltinib for symptomatic tenosynovial giant cell tumor, https://www.fda.gov/drugs/resources-information-approved-drugs/fda-approves-vimseltinib-symptomatic-tenosynovial-giant-cell-tumor

[cit89] Caldwell T. M., Ahn Y. M., Bulfer S. L., Leary C. B., Hood M. M., Lu W.-P., Vogeti L., Vogeti S., Kaufman M. D., Wise S. C., Le Bourdonnec B., Smith B. D., Flynn D. L. (2022). Bioorg. Med. Chem. Lett..

[cit90] Turner N. C., Ro J., André F., Loi S., Verma S., Iwata H., Harbeck N., Loibl S., Huang Bartlett C., Zhang K. (2015). N. Engl. J. Med..

[cit91] Finn R. S., Crown J. P., Lang I., Boer K., Bondarenko I. M., Kulyk S. O., Ettl J., Patel R., Pinter T., Schmidt M., Shparyk Y., Thummala A. R., Voytko N. L., Fowst C., Huang X., Kim S. T., Randolph S., Slamon D. J. (2015). Lancet Oncol..

[cit92] FDA , FDA approves inavolisib with palbociclib and fulvestrant for endocrine-resistant, PIK3CA-mutated, HR-positive, HER2-negative, advanced breast cancer, https://www.fda.gov/drugs/resources-information-approved-drugs/fda-approves-inavolisib-palbociclib-and-fulvestrant-endocrine-resistant-pik3ca-mutated-hr-positive

[cit93] Poratti M., Marzaro G. (2019). Eur. J. Med. Chem..

[cit94] Sledge Jr G. W., Toi M., Neven P., Sohn J., Inoue K., Pivot X., Burdaeva O., Okera M., Masuda N., Kaufman P. A., Koh H., Grischke E. M., Frenzel M., Lin Y., Barriga S., Smith I. C., Bourayou N., Llombart-Cussac A. (2017). J. Clin. Oncol..

[cit95] Goetz M. P., Toi M., Campone M., Sohn J., Paluch-Shimon S., Huober J., Park I. H., Trédan O., Chen S. C., Manso L., Freedman O. C., Garnica Jaliffe G., Forrester T., Frenzel M., Barriga S., Smith I. C., Bourayou N., Di Leo A. (2017). J. Clin. Oncol..

[cit96] FDA , FDA expands early breast cancer indication for abemaciclib with endocrine therapy, https://www.fda.gov/drugs/resources-information-approved-drugs/fda-expands-early-breast-cancer-indication-abemaciclib-endocrine-therapy

[cit97] Shi C., Wang Q., Liao X., Ge H., Huo G., Zhang L., Chen N., Zhai X., Hong Y., Wang L., Han Y., Xiao W., Wang Z., Shi W., Mao Y., Yu J., Xia G., Liu Y. (2019). Eur. J. Med. Chem..

[cit98] Armstrong A. E., Belzberg A. J., Crawford J. R., Hirbe A. C., Wang Z. J. (2023). BMC Cancer.

[cit99] Philippidis A. , Healx Candidate, SpringWorks Therapy Expand NF1 Treatment Options: Healx doses first patient in Phase II trial of HLX-1502 for neurofibromatosis type 1, while SpringWorks wins FDA approval for Gomekli, first drug designed to treat the disease in adults and children, 2025, vol. 7, pp. 136–141

[cit100] FDA , FDA approves mirdametinib for adult and pediatric patients with neurofibromatosis type 1 who have symptomatic plexiform neurofibromas not amenable to complete resection, https://www.fda.gov/drugs/resources-information-approved-drugs/fda-approves-mirdametinib-adult-and-pediatric-patients-neurofibromatosis-type-1-who-have-symptomatic

[cit101] Gross A. M., Wolters P. L., Dombi E., Baldwin A., Whitcomb P., Fisher M. J., Weiss B., Kim A., Bornhorst M., Shah A. C. (2020). N. Engl. J. Med..

[cit102] Hashem O., Shahin A. I., Al Hindawi M. A., Fageeri M. F., Al-Sbbagh S. A., Tarazi H., El-Gamal M. I. (2024). Eur. J. Med. Chem..

[cit103] Hao C., Li X., Wang Z., Liu L., He F., Pan Z. (2023). Eur. J. Med. Chem..

[cit104] Zhang X., Xiao N., Yoshino T., Yang Z., Chen J. (2025). Microenvironment, Overcoming Resistance Mechanisms, and Implementing Biomarker-Guided Combination Treatments. AI Med.

[cit105] Hafner M., Mills C. E., Subramanian K., Chen C., Chung M., Boswell S. A., Everley R. A., Liu C., Walmsley C. S., Juric D., Sorger P. K. (2019). Cell Chem. Biol..

[cit106] Elgawish M. S., Almatary A. M., Zaitone S. A., Salem M. S. H. (2025). Leveraging artificial intelligence and machine learning in kinase inhibitor development: advances, challenges, and future prospects. RSC Med. Chem..

[cit107] Andre T., Berton D., Curigliano G., Jimenez-Rodriguez B., Ellard S., Gravina A., Miller R., Tinker A., Jewell A., Pikiel J. (2022). J. Clin. Oncol..

[cit108] FDA , FDA expands endometrial cancer indication for dostarlimab-gxly with chemotherapy, https://www.fda.gov/drugs/resources-information-approved-drugs/fda-expands-endometrial-cancer-indication-dostarlimab-gxly-chemotherapy

[cit109] Lakhani N., Cosman R., Banerji U., Rasco D., Tomaszewska-Kiecana M., Garralda E., Kornacki D., Li J., Tian C., Bourayou N., Powderly J. (2024). A first-in-human phase I study of the PD-1 inhibitor, retifanlimab (INCMGA00012), in patients with advanced solid tumors (POD1UM-101). ESMO Open.

[cit110] FDA , FDA grants accelerated approval to retifanlimab-dlwr for metastatic or recurrent locally advanced Merkel cell carcinoma, https://www.fda.gov/drugs/resources-information-approved-drugs/fda-grants-accelerated-approval-retifanlimab-dlwr-metastatic-or-recurrent-locally-advanced-merkel

[cit111] Wolchok J. D., Kluger H., Callahan M. K., Postow M. A., Rizvi N. A., Lesokhin A. M., Segal N. H., Ariyan C. E., Gordon R. A., Reed K., Burke M. M., Caldwell A., Kronenberg S. A., Agunwamba B. U., Zhang X., Lowy I., Inzunza H. D., Feely W., Horak C. E., Hong Q., Korman A. J., Wigginton J. M., Gupta A., Sznol M. (2013). N. Engl. J. Med..

[cit112] Larkin J., Chiarion-Sileni V., Gonzalez R., Grob J. J., Rutkowski P., Lao C. D., Cowey C. L., Schadendorf D., Wagstaff J., Dummer R., Ferrucci P. F., Smylie M., Hogg D., Hill A., Márquez-Rodas I., Haanen J., Guidoboni M., Maio M., Schöffski P., Carlino M. S., Lebbé C., McArthur G., Ascierto P. A., Daniels G. A., Long G. V., Bastholt L., Rizzo J. I., Balogh A., Moshyk A., Hodi F. S., Wolchok J. D. (2019). N. Engl. J. Med..

[cit113] Tawbi H. A., Schadendorf D., Lipson E. J., Ascierto P. A., Matamala L., Castillo Gutiérrez E., Rutkowski P., Gogas H. J., Lao C. D., De Menezes J. J. (2022). N. Engl. J. Med..

[cit114] Alkholifi F. K., Alsaffar R. M. (2022). Medicina.

[cit115] Kciuk M., Wanke K., Kruczkowska W., Marciniak B., Kontek R. (2025). Molecules.

[cit116] Gupta S., Wang C., Raetz E. A., Schore R., Salzer W. L., Larsen E. C., Maloney K. W., Mattano L. A., Carroll W. L., Winick N. J., Hunger S. P., Loh M. L., Devidas M. (2020). J. Clin. Oncol..

[cit117] Lin D. Y., Andreotti A. H. (2023). PLoS One.

[cit118] Kurtzberg J., Abdel-Azim H., Carpenter P., Chaudhury S., Horn B., Mahadeo K., Nemecek E., Neudorf S., Prasad V., Prockop S. (2020). Biol. Blood Marrow Transplant..

[cit119] Mahat U., Przepiorka D., Lola A. F.-A. (2025). Remestemcel-L-rknd for Steroid-Refractory Acute Graft-vs-Host Disease in Pediatric Patients. JAMA.

[cit120] FDA , FDA approves remestemcel-L-rknd for steroid-refractory acute graft versus host disease in pediatric patients, https://www.fda.gov/drugs/resources-information-approved-drugs/fda-approves-remestemcel-l-rknd-steroid-refractory-acute-graft-versus-host-disease-pediatric 10.1001/jama.2025.6179 40397429

[cit121] Bako L., Cornish K. W., Hill L. L., Ivetic A., Srivastava D., Dazzi F., Burns C. (2025). Cytotherapy.

[cit122] Giebel B. , Extracellular Vesicles and Circulating Nucleic Acids, 2025, vol. 6, p. 183 10.20517/evcna.2025.02 PMC11977348 40206802

[cit123] Bi C., Patel J. S., Liang S. H. (2025). J. Med. Chem..

[cit124] Poumeaud F., Morisseau M., Cabel L., Gonçalves A., Rivier C., Trédan O., Volant E., Frenel J. S., Ladoire S., Jacot W., Jamelot M., Foka Tichoue H., Patsouris A., Teixeira L., Bidard F. C., Loirat D., Brunet M., Levy C., Bailleux C., Cabarrou B., Deleuze A., Uwer L., Deluche E., Grellety T., Franchet C., Fiteni F., Bischoff H., Vion R., Pagliuca M., Verret B., Bécourt S., Reverdy T., de Nonneville A., Dalenc F. (2024). Br. J. Cancer.

[cit125] Bouziane J., Loap P., Cao K., Allali S., Gounane Y., Loganadane G., Escalup L., Pierga J. Y., Kirova Y. (2024). Am. J. Clin. Oncol..

[cit126] FDA , FDA approves fam-trastuzumab deruxtecan-nxki for unresectable or metastatic HR-positive, HER2-low or HER2-ultralow breast cancer, https://www.fda.gov/drugs/resources-information-approved-drugs/fda-approves-fam-trastuzumab-deruxtecan-nxki-unresectable-or-metastatic-hr-positive-her2-low-or-her2

[cit127] Jang J. Y., Kim D., Lee N. K., Im E., Kim N. D. (2025). Int. J. Mol. Sci..

[cit128] Matikonda S. S., McLaughlin R., Shrestha P., Lipshultz C., Schnermann M. J. (2022). Bioconjug. Chem..

[cit129] Okajima D., Yasuda S., Maejima T., Karibe T., Sakurai K., Aida T., Toki T., Yamaguchi J., Kitamura M., Kamei R., Fujitani T., Honda T., Shibutani T., Muramatsu S., Nakada T., Goto R., Takahashi S., Yamaguchi M., Hamada H., Noguchi Y., Murakami M., Abe Y., Agatsuma T. (2021). Mol. Cancer Therapeut..

[cit130] Parekh D., Desai D., Sampat P. J., Abhyankar A. V., Paulraj S., Shah A., Ashok Kumar P. (2025). J. Clin. Oncol..

[cit131] FDA , FDA approves datopotamab deruxtecan-dlnk for unresectable or metastatic, HR-positive, HER2-negative breast cancer, https://www.fda.gov/drugs/resources-information-approved-drugs/fda-approves-datopotamab-deruxtecan-dlnk-unresectable-or-metastatic-hr-positive-her2-negative-breast 10.1158/1078-0432.CCR-25-1388 PMC12393668 40864501

[cit132] Meric-Bernstam F., Yuca E., Evans K. W., Zhao M., Maejima T., Karibe T., Raso M. G., Tang X., Zheng X., Rizvi Y. Q., Akcakanat A., Scott S. M., Wang B., Byers L. A., Tripathy D., Okajima D., Damodaran S. (2025). Clin. Cancer Res..

[cit133] Sawant S., Naik G. G., Sahu A. N., Jagtap V. A. (2024). Med. Oncol..

[cit134] Mer A. H., Mirzaei Y., Misamogooe F., Bagheri N., Bazyari A., Keshtkaran Z., Meyfour A., Shahedi A., Amirkhani Z., Jafari A. (2024). Drug Deliv. Transl. Res..

[cit135] FDA , FDA grants accelerated approval to telisotuzumab vedotin-tllv for NSCLC with high c-Met protein overexpression, https://www.fda.gov/drugs/resources-information-approved-drugs/fda-grants-accelerated-approval-telisotuzumab-vedotin-tllv-nsclc-high-c-met-protein-overexpression

[cit136] Schreiber A. R., O'Bryant C. L., Kabos P., Diamond J. R. (2023). Expet Rev. Anticancer Ther..

[cit137] Główka F., Kasprzyk A., Romański M., Wróbel T., Wachowiak J., Szpecht D., Kałwak K., Wiela-Hojeńska A., Dziatkiewicz P., Teżyk A., Żaba C. (2015). Eur. J. Pharm. Sci..

[cit138] O‘Hagan Henderson S., Frietsch J. J., Hilgendorf I., Hochhaus A., Köhne C.-H., Casper J. (2022). J. Cancer Res. Clin. Oncol..

[cit139] FDA , FDA approves treosulfan with fludarabine as a preparative regimen for alloHSCT in adult and pediatric patients with AML or MDS, https://www.fda.gov/drugs/resources-information-approved-drugs/fda-approves-treosulfan-fludarabine-preparative-regimen-allohsct-adult-and-pediatric-patients-aml-or

[cit140] Romański M., Wachowiak J., Główka F. K. (2018). Clin. Pharmacokinet..

[cit141] Westerhof G. R., Ploemacher R. E., Boudewijn A., Blokland I., Dillingh J. H., McGown A. T., Hadfield J. A., Dawson M. J., Down J. D. (2000). Cancer Res..

[cit142] Yaeger R., Langer C. J., Ruffinelli J. C., Fakih M., Furqan M., Machiels J.-P. H., Saportas Y., Nolte-Hippenmeyer J., Chan E., Xia C., Masuishi T. (2024). J. Clin. Oncol..

[cit143] Fakih M. G., Salvatore L., Esaki T., Modest D. P., Lopez-Bravo D. P., Taieb J., Karamouzis M. V., Ruiz-Garcia E., Kim T. W., Kuboki Y., Meriggi F., Cunningham D., Yeh K. H., Chan E., Chao J., Saportas Y., Tran Q., Cremolini C., Pietrantonio F. (2023). N. Engl. J. Med..

[cit144] FDA , FDA approves sotorasib with panitumumab for KRAS G12C-mutated colorectal cancer, https://www.fda.gov/drugs/resources-information-approved-drugs/fda-approves-sotorasib-panitumumab-kras-g12c-mutated-colorectal-cancer

[cit145] Canon J., Rex K., Saiki A. Y., Mohr C., Cooke K., Bagal D., Gaida K., Holt T., Knutson C. G., Koppada N., Lanman B. A., Werner J., Rapaport A. S., San Miguel T., Ortiz R., Osgood T., Sun J.-R., Zhu X., McCarter J. D., Volak L. P., Houk B. E., Fakih M. G., O'Neil B. H., Price T. J., Falchook G. S., Desai J., Kuo J., Govindan R., Hong D. S., Ouyang W., Henary H., Arvedson T., Cee V. J., Lipford J. R. (2019). Nature.

[cit146] Pandey D., Chauhan S. C., Kashyap V. K., Roy K. K. (2024). Eur. J. Med. Chem..

[cit147] Rubinson D. A., Tanaka N., Fece de la Cruz F., Kapner K. S., Rosenthal M. H., Norden B. L., Barnes H., Ehnstrom S., Morales-Giron A. A., Brais L. K. (2024). Cancer Discov..

[cit148] Kwan A. K., Piazza G. A., Keeton A. B., Leite C. A. (2022). J. Exp. Clin. Cancer Res..

[cit149] Kopetz S., Yoshino T., Van Cutsem E., Eng C., Kim T. W., Wasan H. S., Desai J., Ciardiello F., Yaeger R., Maughan T. S., Beyzarov E., Zhang X., Ferrier G., Zhang X., Tabernero J. (2025). Nat. Med..

[cit150] Aboubakar Nana F., Ocak S. (2021). Pharmaceutics.

[cit151] FDA , FDA grants accelerated approval to encorafenib with cetuximab and mFOLFOX6 for metastatic colorectal cancer with a BRAF V600E mutation, https://www.fda.gov/drugs/resources-information-approved-drugs/fda-grants-accelerated-approval-encorafenib-cetuximab-and-mfolfox6-metastatic-colorectal-cancer-braf

[cit152] Kopetz S., Grothey A., Yaeger R., Van Cutsem E., Desai J., Yoshino T., Wasan H., Ciardiello F., Loupakis F., Hong Yong S., Steeghs N., Guren Tormod K., Arkenau H.-T., Garcia-Alfonso P., Pfeiffer P., Orlov S., Lonardi S., Elez E., Kim T.-W., Schellens Jan H. M., Guo C., Krishnan A., Dekervel J., Morris V., Calvo Ferrandiz A., Tarpgaard L. S., Braun M., Gollerkeri A., Keir C., Maharry K., Pickard M., Christy-Bittel J., Anderson L., Sandor V., Tabernero J. (2019). N. Engl. J. Med..

[cit153] Gamal El-Din M. M., El-Gamal M. I., Abdel-Maksoud M. S., Yoo K. H., Baek D., Choi J., Lee H., Oh C.-H. (2016). J. Enzyme Inhib. Med. Chem..

[cit154] Byrd J. C., Hillmen P., Ghia P., Kater A. P., Chanan-Khan A., Furman R. R., O'Brien S., Yenerel M. N., Illés A., Kay N., Garcia-Marco J. A., Mato A., Pinilla-Ibarz J., Seymour J. F., Lepretre S., Stilgenbauer S., Robak T., Rothbaum W., Izumi R., Hamdy A., Patel P., Higgins K., Sohoni S., Jurczak W. (2021). J. Clin. Oncol..

[cit155] Wang M., Salek D., Belada D., Song Y., Jurczak W., Kahl B. S., Paludo J., Chu M. P., Kryachok I., Fogliatto L., Cheah C. Y., Morawska M., Sancho J.-M., Li Y., Patti C., Forsyth C., Zhang J., Lesley R., Ramadan S., Rule S., Dreyling M. (2025). J. Clin. Oncol..

[cit156] FDA , FDA approves acalabrutinib with bendamustine and rituximab for previously untreated mantle cell lymphoma, https://www.fda.gov/drugs/resources-information-approved-drugs/fda-approves-acalabrutinib-bendamustine-and-rituximab-previously-untreated-mantle-cell-lymphoma

[cit157] Silverman J. E. Y. (2016). ACS Med. Chem. Lett..

[cit158] Byrd John C., Harrington B., O'Brien S., Jones Jeffrey A., Schuh A., Devereux S., Chaves J., Wierda William G., Awan Farrukh T., Brown Jennifer R., Hillmen P., Stephens Deborah M., Ghia P., Barrientos Jacqueline C., Pagel John M., Woyach J., Johnson D., Huang J., Wang X., Kaptein A., Lannutti Brian J., Covey T., Fardis M., McGreivy J., Hamdy A., Rothbaum W., Izumi R., Diacovo Thomas G., Johnson Amy J., Furman Richard R. (2016). N. Engl. J. Med..

[cit159] Wu J., Zhang M., Liu D. (2016). J. Hematol. Oncol..

[cit160] Hallin J., Engstrom L. D., Hargis L., Calinisan A., Aranda R., Briere D. M., Sudhakar N., Bowcut V., Baer B. R., Ballard J. A. (2020). Cancer Discov..

[cit161] Kopetz S., Grothey A., Yaeger R., Van Cutsem E., Desai J., Yoshino T., Wasan H., Ciardiello F., Loupakis F., Hong Y. S. (2019). N. Engl. J. Med..

[cit162] Grisham R., Monk B. J., Van Nieuwenhuysen E., Moore K. N., Fabbro M., O'Malley D. M., Oaknin A., Thaker P., Oza A. M., Colombo N., Gershenson D., Aghajanian C. A., Choi C. H., Lee Y. C., Mirza M. R., Coleman R. L., Cobb L., Harter P., Lustgarten S., Youssoufian H., Banerjee S. (2025). Int. J. Gynecol. Cancer.

[cit163] Dawson A. , Targeted Therapy in Low-Grade Serous Ovarian Carcinoma: Characterization of MEK Inhibitor Response in Novel Patient-Derived Cell Lines, University of British Columbia, 2016

[cit164] FDA , FDA grants accelerated approval to the combination of avutometinib and defactinib for KRAS-mutated recurrent low-grade serous ovarian cancer, https://www.fda.gov/drugs/resources-information-approved-drugs/fda-grants-accelerated-approval-combination-avutometinib-and-defactinib-kras-mutated-recurrent-low 10.1080/14737140.2026.2665840 42059499

[cit165] Ram T., Singh A. K., Kumar A., Singh H., Pathak P., Grishina M., Khalilullah H., Jaremko M., Emwas A.-H., Verma A., Kumar P. (2023). RSC Med. Chem..

[cit166] Zhu S.-K., Wu Q., Liu G.-X., Geng A.-Q., Wang P.-A. (2025). RSC Adv..

[cit167] Wang S., Zhang R.-H., Zhang H., Wang Y.-C., Yang D., Zhao Y.-L., Yan G.-Y., Xu G.-B., Guan H.-Y., Zhou Y.-H., Cui D.-B., Liu T., Li Y.-J., Liao S.-G., Zhou M. (2021). Eur. J. Med. Chem..

[cit168] Gelderblom H., Razak A. A., Taylor M. H., Bauer T. M., Wilky B., Martin-Broto J., Gonzalez A. F., Rutkowski P., Szostakowski B., Alcindor T., Saleh R., Genta S., Stacchiotti S., van de Sande M., Wagner A. J., Bernthal N., Davis L. E., Vuky J., Tait C., Matin B., Narasimhan S., Sharma M. G., Ruiz-Soto R., Sherman M. L., Tap W. D. (2024). Clin. Cancer Res..

[cit169] Smith B. D., Kaufman M. D., Wise S. C., Ahn Y. M., Caldwell T. M., Leary C. B., Lu W.-P., Tan G., Vogeti L., Vogeti S. (2021). Mol. Cancer Therapeut..

[cit170] Hoy S. M. (2025). Mirdametinib: First Approval. Drugs.

[cit171] de Blank P. M. K., Gross A. M., Akshintala S., Blakeley J. O., Bollag G., Cannon A., Dombi E., Fangusaro J., Gelb B. D., Hargrave D., Kim A., Klesse L. J., Loh M., Martin S., Moertel C., Packer R., Payne J. M., Rauen K. A., Rios J. J., Robison N., Schorry E. K., Shannon K., Stevenson D. A., Stieglitz E., Ullrich N. J., Walsh K. S., Weiss B. D., Wolters P. L., Yohay K., Yohe M. E., Widemann B. C., Fisher M. J. (2022). Neuro Oncol..

[cit172] Li C., Sun Y.-D., Yu G.-Y., Cui J.-R., Lou Z., Zhang H., Huang Y., Bai C.-G., Deng L.-L., Liu P., Zheng K., Wang Y.-H., Wang Q.-Q., Li Q.-R., Wu Q.-Q., Liu Q., Shyr Y., Li Y.-X., Chen L.-N., Wu J.-R., Zhang W., Zeng R. (2020). Cancer Cell.

[cit173] An D., Banerjee S., Lee J.-M. (2021). Cancer Treat Rev..

[cit174] Primeau A. S. B. , Divarasib Shows Activity in Patients With KRAS-Mutated Solid Tumors, Cancer Therapy Advisor, 2023

[cit175] Morstein J., Bowcut V., Fernando M., Yang Y., Zhu L., Jenkins M. L., Evans J. T., Guiley K. Z., Peacock D. M., Krahnke S., Lin Z., Taran K. A., Huang B. J., Stephen A. G., Burke J. E., Lightstone F. C., Shokat K. M. (2024). Cell.

[cit176] Fernando M. C., Craven G. B., Shokat K. M. (2024). Small GTPases.

[cit177] Shi Y., Fang J., Xing L., Yao Y., Zhang J., Liu L., Wang Y., Hu C., Xiong J., Liu Z., Yang R., Wang Z., Zhao E., Wang M., Zhao Y., Tang K., Li Z., Song Z. (2024). J. Clin. Oncol..

[cit178] Li A., Li S., Wang P., Dang C., Fan X., Chen M., Liu D., Li F., Liu H., Zhang W., Wang Y., Wang Y. (2025). J. Med. Chem..

[cit179] Wang P., Sun X., He X., Kang D., Liu X., Liu D., Li A., Yang G., Lin Y., Li S., Wang Y., Wang Y. (2025). Cancer Res. Commun..

[cit180] Bewersdorf J. P., Mi X., Lu B., Kuykendall A., Sallman D., Patel M., Stevens D., Philipovskiy A., Sutamtewagul G., Masarova L., Keiffer G., Verma A., Bhagwat N., Wang M., Moore A., Rager J., Heiser D., Ro S., Hong W.-J., Abdel-Wahab O., Stein E. M. (2025). Leukemia.

[cit181] Hu M., Chen X. (2024). RSC Adv..

[cit182] Chan-Penebre E., Kuplast K. G., Majer C. R., Boriack-Sjodin P. A., Wigle T. J., Johnston L. D., Rioux N., Munchhof M. J., Jin L., Jacques S. L., West K. A., Lingaraj T., Stickland K., Ribich S. A., Raimondi A., Scott M. P., Waters N. J., Pollock R. M., Smith J. J., Barbash O., Pappalardi M., Ho T. F., Nurse K., Oza K. P., Gallagher K. T., Kruger R., Moyer M. P., Copeland R. A., Chesworth R., Duncan K. W. (2015). Nat. Chem. Biol..

[cit183] Bewersdorf J. P., Mi X., Lu B., Kuykendall A., Sallman D., Patel M., Stevens D., Philipovskiy A., Sutamtewagul G., Masarova L., Keiffer G., Verma A., Bhagwat N., Wang M., Moore A., Rager J., Heiser D., Ro S., Hong W. J., Abdel-Wahab O., Stein E. M. (2025). Leukemia.

[cit184] Drescher C., Walter H. S., Gastinne T., Lakhani N. J., Ribrag V., Rasco D. W., Gutierrez M., Sullivan R. J., Harvey R. D., Banda K., Kwiatek M., Garcia-Sancho A. M., Duska L. R., Zinzani P. L., Thakur A., Kann L., Faulhaber N., Jauch-Lembach J., Kindler H. L. (2023). J. Clin. Oncol..

[cit185] Xia J., Li J., Tian L., Ren X., Liu C., Liang C. (2022). J. Med. Chem..

[cit186] Keller P. J., Adams E. J., Wu R., Côté A., Arora S., Cantone N., Meyer R., Mertz J. A., Gehling V., Cui J., Stuckey J. I., Khanna A., Zhao F., Chen Z., Yu Z., Cummings R. T., Taimi M., Lakhani N. J., Rasco D., Gutierrez M., Duska L., Devitt M., Rippley R., Levell J., Truong J., Wang J., Sun K., Trojer P. (2024). Cancer Res..

[cit187] Delage B., Fennell D. A., Nicholson L., McNeish I., Lemoine N. R., Crook T., Szlosarek P. W. (2010). Int. J. Cancer.

[cit188] Schwarz R., Zitzow E., Fiebig A., Hering S., Humboldt Y., Schoenwaelder N., Kämpfer N., Volkmar K., Hinz B., Kreikemeyer B. (2022). Appl. Microbiol. Biotechnol..

[cit189] Miraki-Moud F., Ghazaly E., Ariza-McNaughton L., Hodby K. A., Clear A., Anjos-Afonso F., Liapis K., Grantham M., Sohrabi F., Cavenagh J. J. B. (2015). J. Am. Soc. Hematol..

[cit190] Ayzman A., Pachynski R. K., Reimers M. A. (2025). Curr. Treat. Options Oncol..

[cit191] Kratochwil C., Bruchertseifer F., Giesel F. L., Weis M., Verburg F. A., Mottaghy F., Kopka K., Apostolidis C., Haberkorn U., Morgenstern A. (2016). J. Nucl. Med..

[cit192] Bidkar A. P., Zerefa L., Yadav S., VanBrocklin H. F., Flavell R. R. (2024). Theranostics.

[cit193] Schäfer M., Bauder-Wüst U., Roscher M., Motlová L., Kutilová Z., Remde Y., Klika K. D., Graf J., Bařinka C., Benešová-Schäfer M. (2025). ACS Omega.

[cit194] Goodfellow R. (2015). Immunotherapy.

[cit195] Patel P. M., Ottensmeier C. H., Mulatero C., Lorigan P., Plummer R., Pandha H., Elsheikh S., Hadjimichael E., Villasanti N., Adams S. E., Cunnell M., Metheringham R. L., Brentville V. A., Machado L., Daniels I., Gijon M., Hannaman D., Durrant L. G. (2018). OncoImmunology.

[cit196] Irie H., Morita K., Matsuda M., Koizumi M., Mochizuki S. (2023). Bioconjug. Chem..

[cit197] Bhole R. P., Labhade S., Gurav S. S. (2025). Bioanalysis.

[cit198] He S., Dong G., Cheng J., Wu Y., Sheng C. (2022). Med. Res. Rev..

[cit199] Sharma J., Keeling K. M., Rowe S. M. (2020). Eur. J. Med. Chem..

[cit200] Maines L. W., Keller S. N., Smith C. D. Int. J. Mol. Sci..

[cit201] Tawati S. M. , Design, synthesis and biological evaluation of sphingosine kinase inhibitors for the treatment of prostate cancer, 2018, https://stax.strath.ac.uk/concern/theses/js956f85s

[cit202] Baek D. J., MacRitchie N., Anthony N. G., Mackay S. P., Pyne S., Pyne N. J., Bittman R. (2013). J. Med. Chem..

[cit203] Pashikanti S., Foster D. J., Kharel Y., Brown A. M., Bevan D. R., Lynch K. R., Santos W. L. (2022). ACS Bio Med Chem Au.

[cit204] Garcia-Manero G., Luger S., Venugopal P., Maness L., Wetzler M., Coutre S., Stock W., Borthakur G., Chiao J., Kantarjian H. (2009). J. Clin. Oncol..

[cit205] Khan M. F., Machuca M. A., Rahman M. M., Koç C., Norton R. S., Smith B. J., Roujeinikova A. (2020). Biomolecules.

[cit206] Liu X., Kantarjian H., Plunkett W. (2012). Expet Opin. Invest. Drugs.

[cit207] Li J., Haynes N., Cummins K., Gowrishankar K., Micklewaithe K., Hilton H., Dunn R., Oliaro J., Harrison S. (2023). Cancer Res..

[cit208] Ohlstrom D., Walker Z. J., Pandey A., Davis L. N., Engel K. L., Pan Z., Forsberg P. A., Mark T. M., Gillen A. E., Sherbenou D. W. (2025). Cancer Res. Commun..

[cit209] Hong D. S., Cappuzzo F., Chul Cho B., Dowlati A., Hussein M., Kim D.-W., Percent I., Christensen J. G., Morin J., Potvin D., Faltaos D., Tassell V., Der-Torossian H., Chao R. (2024). Lung Cancer.

[cit210] Zhang H., Zhijun S., Zhaopeng L., Song C. (2018). Future Med. Chem..

[cit211] Engstrom L. D., Aranda R., Lee M., Tovar E. A., Essenburg C. J., Madaj Z., Chiang H., Briere D., Hallin J., Lopez-Casas P. P., Baños N., Menendez C., Hidalgo M., Tassell V., Chao R., Chudova D. I., Lanman R. B., Olson P., Bazhenova L., Patel S. P., Graveel C., Nishino M., Shapiro G. I., Peled N., Awad M. M., Jänne P. A., Christensen J. G. (2017). Clin. Cancer Res..

[cit212] Jain K. K. , in Textbook of Personalized Medicine, ed. K. K. Jain, Springer New York, New York, NY, 2015, pp. 199–381, 10.1007/978-1-4939-2553-7_10

[cit213] Castaigne S., Pautas C., Terré C., Raffoux E., Bordessoule D., Bastie J.-N., Legrand O., Thomas X., Turlure P., Reman O., de Revel T., Gastaud L., de Gunzburg N., Contentin N., Henry E., Marolleau J.-P., Aljijakli A., Rousselot P., Fenaux P., Preudhomme C., Chevret S., Dombret H. (2012). Lancet.

[cit214] Savoy E. A. , Olatunji F. P., Yoon H., Mesbahi N., Knight J. R. and Berkman C. E., Acid-labile linkers, Chemical Linkers in Antibody–Drug Conjugates (ADCs), 2021, pp. 213–231, 10.1039/9781839165153-00213

[cit215] Bross P. F., Beitz J., Chen G., Chen X. H., Duffy E., Kieffer L., Roy S., Sridhara R., Rahman A., Williams G., Pazdur R. (2001). Clin. Cancer Res..

[cit216] Han Y. C., Kahler J., Piché-Nicholas N., Hu W., Thibault S., Jiang F., Leal M., Katragadda M., Maderna A., Dushin R., Prashad N., Charati M. B., Clark T., Tumey L. N., Tan X., Giannakou A., Rosfjord E., Gerber H. P., Tchistiakova L., Loganzo F., O'Donnell C. J., Sapra P. (2021). Clin. Cancer Res..

[cit217] Rodriguez-Otero P., Tamariz L. E., San-Miguel J. F. (2023). Lancet Haematol..

[cit218] Baines A. C., Ershler R., Kanapuru B., Xu Q., Shen G., Li L., Ma L., Okusanya O. O., Simpson N. E., Nguyen W., Theoret M. R., Pazdur R., Gormley N. J. (2022). Clin. Cancer Res..

[cit219] Demel I., Bago J. R., Hajek R., Jelinek T. (2021). Br. J. Haematol..

[cit220] Powles T., Tagawa S., Vulsteke C., Gross-Goupil M., Park S. H., Necchi A., De Santis M., Duran I., Morales-Barrera R., Guo J., Sternberg C. N., Bellmunt J., Goebell P. J., Kovalenko M., Boateng F., Sierecki M., Wang L., Sima C. S., Waldes J., Loriot Y., Grivas P. (2025). Ann. Oncol..

[cit221] Zhang M., Zuo Y., Chen S., Li Y., Xing Y., Yang L., Wang H., Guo R. (2024). Front. Oncol..

[cit222] Liu X., Ma L., Li J., Sun L., Yang Y., Liu T., Xing D., Yan S., Zhang M. (2024). Theranostics.

[cit223] Horwitz S. M., Hamadani M., Fanale M. A., Feingold J., Spira A. I., Fields P. A., Menne T. (2017). *et al.*, Interim results from a phase 1 study of ADCT-301 (camidanlumab tesirine) show promising activity of a novel pyrrolobenzodiazepine-based antibody drug conjugate in relapsed/refractory Hodgkin/non-Hodgkin lymphoma. Blood.

[cit224] Hartley J. A. (2021). Expet Opin. Biol. Ther..

[cit225] Murer P., Brannetti B., Rondeau J. M., Petersen L., Egli N., Popp S., Regnier C., Richter K., Katopodis A., Huber C. (2024). mAbs.

[cit226] Thomson C., Barton P., Braybrooke E., Colclough N., Dong Z., Evans L., Floc’h N., Guérot C., Hargreaves D., Khurana P. (2024). J. Med. Chem..

[cit227] FDA US , FDA grants accelerated approval to mobocertinib for metastatic non-small cell lung cancer with EGFR exon 20 insertion mutations, in Drugs/Approved Drugs, US Food & Drug Administration, Silver Spring, MD, 2021

[cit228] Wang J., Lam D., Yang J., Hu L. (2022). Med. Chem. Res..

[cit229] Huang W.-S., Li F., Gong Y., Zhang Y., Youngsaye W., Xu Y., Zhu X., Greenfield M. T., Kohlmann A., Taslimi P. M., Toms A., Zech S. G., Zhou T., Das B., Jang H. G., Tugnait M., Ye Y. E., Gonzalvez F., Baker T. E., Nadworny S., Ning Y., Wardwell S. D., Zhang S., Gould A. E., Hu Y., Lane W., Skene R. J., Zou H., Clackson T., Narasimhan N. I., Rivera V. M., Dalgarno D. C., Shakespeare W. C. (2023). Bioorg. Med. Chem. Lett..

[cit230] FDA , WITHDRAWN: FDA grants accelerated approval to infigratinib for metastatic cholangiocarcinoma, https://www.fda.gov/drugs/resources-information-approved-drugs/withdrawn-fda-grants-accelerated-approval-infigratinib-metastatic-cholangiocarcinoma

[cit231] Abou-Alfa G. K., Borbath I., Roychowdhury S., Goyal L., Lamarca A., Macarulla T., Shroff R. T., Oh D.-Y., Javle M. M., Tamas C., Savastano D. M., Van Veenhuyzen D. F., Xu C., Solanas J., Freas E. (2024). J. Clin. Oncol..

[cit232] Subbiah V., Verstovsek S. (2023). Cell reports. Medicine.

[cit233] Choi K. (2023). Curr. Med. Chem..

[cit234] Guagnano V., Furet P., Spanka C., Bordas V., Le Douget M., Stamm C., Brueggen J., Jensen M. R., Schnell C., Schmid H., Wartmann M., Berghausen J., Drueckes P., Zimmerlin A., Bussiere D., Murray J., Graus Porta D. (2011). J. Med. Chem..

[cit235] Zou L., Qi Y., Tang L., Du Y., Xiang M., Chen X., Ma J., Yang Z. (2022). Chin. J. Cancer Res..

[cit236] Scott W. J., Hentemann M. F., Rowley R. B., Bull C. O., Jenkins S., Bullion A. M., Johnson J., Redman A., Robbins A. H., Esler W., Fracasso R. P., Garrison T., Hamilton M., Michels M., Wood J. E., Wilkie D. P., Xiao H., Levy J., Stasik E., Liu N., Schaefer M., Brands M., Lefranc J. (2016). ChemMedChem.

[cit237] Krause G., Hassenrück F., Hallek M. (2018). Drug Des. Dev. Ther..

[cit238] Schjesvold F. H., Dimopoulos M.-A., Delimpasi S., Robak P., Coriu D., Legiec W., Pour L., Špička I., Masszi T., Doronin V., Minarik J., Salogub G., Alekseeva Y., Lazzaro A., Maisnar V., Mikala G., Rosiñol L., Liberati A. M., Symeonidis A., Moody V., Thuresson M., Byrne C., Harmenberg J., Bakker N. A., Hájek R., Mateos M.-V., Richardson P. G., Sonneveld P., Schjesvold F., Delimpasi S., Robak P., Coriu D., Nikolayeva A., Tomczak W., Pour L., Spicka I., Dimopoulos M.-A., Masszi T., Doronin V., Minarik J., Salogub G., Alekseeva Y., Maisnar V., Mikala G., Rosinol L., Konstantinova T., Lazzaro A., Liberati A. M., Symeonidis A., Gatt M., Illes A., Abdulhaq H., Dungarwalla M., Grosicki S., Hajek R., Leleu X., Myasnikov A., Richardson P. G., Avivi I., Deeren D., Gironella M., Hernandez-Garcia M. T., Martinez Lopez J., Newinger-Porte M., Ribas P., Samoilova O., Voog E., Arnao-Herraiz M., Carrillo-Cruz E., Corradini P., Dodlapati J., Granell Gorrochategui M., Huang S.-Y., Jenner M., Karlin L., Kim J. S., Kopacz A., Medvedeva N., Min C.-K., Mina R., Palk K., Shin H.-J., Sohn S. K., Sonneveld P., Tache J., Anagnostopoulos A., Arguiñano J.-M., Cavo M., Filicko J., Garnes M., Halka J., Herzog-Tzarfati K., Ipatova N., Kim K., Krauth M.-T., Kryuchkova I., Lazaroiu M. C., Luppi M., Proydakov A., Rambaldi A., Rudzianskiene M., Yeh S.-P., Alcalá-Peña M. M., Alegre Amor A., Alizadeh H., Bendandi M., Brearton G., Brown R., Cavet J., Dally N., Egyed M., Hernández-Rivas J. Á., Kaare A., Karsenti J.-M., Kloczko J., Kreisle W., Lee J.-J., Legiec W., Machherndl-Spandl S., Manda S., Mateos M.-V., Moiseev I., Moreb J., Nagy Z., Nair S., Oriol-Rocafiguera A., Osswald M., Otero-Rodriguez P., Peceliunas V., Plesner T., Rey P., Rossi G., Stevens D., Suriu C., Tarella C., Verlinden A., Zannetti A. (2022). Lancet Haematol..

[cit239] Pahwa R., Chhabra J., Kumar R., Narang R. (2022). Eur. J. Med. Chem..

[cit240] Chauhan D., Ray A., Viktorsson K., Spira J., Paba-Prada C., Munshi N., Richardson P., Lewensohn R., Anderson K. C. (2013). Clin. Cancer Res..

[cit241] FDA , Withdrawn | Cancer Accelerated Approvals, https://www.fda.gov/drugs/resources-information-approved-drugs/withdrawn-cancer-accelerated-approvals

[cit242] Becker S., Kiecke C., Schäfer E., Sinzig U., Deuper L., Trigo-Mourino P., Griesinger C., Koch R., Rydzynska Z., Chapuy B., von Bonin F., Kube D., Venkataramani V., Bohnenberger H., Leha A., Flach J., Dierks S., Bastians H., Maruschak B., Bojarczuk K., Taveira M. O., Trümper L., Wulf G. M., Wulf G. G. (2020). Mol. Cancer Res..

[cit243] Silverman J. A., Deitcher S. R. (2013). Cancer Chemother. Pharmacol..

[cit244] Hellmann M. , Cho B., Juergens R., Cheng Y., de Castro G., Erman M., Bauman J., Takahashi T., Schwarzenberger P. and Zhang P., Regular and Young Investigator Award Abstracts, 2021, vol. 9, pp. A488

[cit245] Takei J., Asano T., Nanamiya R., Nakamura T., Yanaka M., Hosono H., Tanaka T., Sano M., Kaneko M. K., Harada H., Kato Y. (2021). Monoclon. Antibodies Immunodiagn. Immunother..

[cit246] Lavie D., Timmerman J., García-Sanz R., Kim W. S., Kim T. M., Avigdor A., Dierickx D., Jagadeesh D., Molin D. L., Ozcan M., Gokmen Sevindik O., Saeed H., Sidi Y., Pillai P., Marinello P., Herrera A. F. (2023). Blood.

[cit247] Dai T., Sun H., Liban T., Vicente-Suarez I., Zhang B., Song Y., Jiang Z., Yu J., Sheng J., Lv B. (2024). Sci. Rep..

[cit248] Buyukgolcigezli I., Tenekeci A. K., Sahin I. H. (2025). Cancers.

[cit249] Cohen P., Cross D., Jänne P. A. (2021). Nat. Rev. Drug Discovery.

[cit250] Zhong L., Li Y., Xiong L., Wang W., Wu M., Yuan T., Yang W., Tian C., Miao Z., Wang T., Yang S. (2021). Signal Transduct. Targeted Ther..

[cit251] Selvakumar P. , Prabakaran M. and Bhattacharya S., in Spatially Variable Genes in Cancer: Development, Progression, and Treatment Response, IGI Global Scientific Publishing, 2025, pp. 429–446

[cit252] Hingorani D. V. (2024). Expet Opin. Biol. Ther..

[cit253] Wynn C., Patel R., Hillegass W. B., Tang S.-C. (2022). J. Clin. Oncol..

[cit254] Conilh L., Fournet G., Fourmaux E., Murcia A., Matera E. L., Joseph B., Dumontet C., Viricel W. (2021). Pharmaceuticals.

[cit255] Shivani T. A., Rahaman A., Chaudhary S. (2024). Drug discovery today.

[cit256] Hu C., Li S., Yang C., Chen J., Xiong Y., Fan G., Liu H., Hong L. (2023). J. Cheminf..

[cit257] Mishra A., Thakur A., Sharma R., Onuku R., Kaur C., Liou J. P., Hsu S. P., Nepali K. (2024). Expet Opin. Drug Discov..

[cit258] Okafor C. E., Egwuatu E. C., Owosagba V. A., Njei T., Adeyemi B. I., Onuche P. U. O., Adams A., Ugwuja C. B., Chibueze E. S., Lawal O. P. (2025). J. Cancer Tumor Int..

[cit259] Li K., Crews C. M. (2022). Chem. Soc. Rev..

[cit260] Cecchini C., Tardy S., Scapozza L. (2022). Chimia.

[cit261] Zhang X., Song Z., Zhang X., Zou J., Yu G., Chen X., Wang S. (2025). Angew. Chem..

[cit262] Zhang Z. , Expanding the Horizon of Proteolysis Targeting Chimeras (PROTACs) in RAPID Platforms, CLIPTAC, and the Exploration of E3 Ligase Substrate Receptors, PhD dissertation, University of Wisconsin–Madison, 2023

[cit263] Gao H., Sun X., Rao Y. (2020). ACS Med. Chem. Lett..

[cit264] Békés M., Langley D. R., Crews C. M. (2022). Nat. Rev. Drug Discov..

