# Supplementary material for: Medicinal chemistry perspectives on anticancer drug design based on clinical applications (2015–2025)
Source: RSC Adv. 2025 Oct 1;15(43):36441–71. doi: 10.1039/d5ra05472a (PMC12486241; doi:10.1039/d5ra05472a)
Supplement: RA-015-D5RA05472A-s001 [file RA-015-D5RA05472A-s001.pdf]

Supplementary Information (SI) for RSC Advances.
This journal is © The Royal Society of Chemistry 2025

Medicinal Chemistry Perspectives on Anticancer Drug Design based on Clinical Applications (2015-2025)

Ahmed A. Al-Karmalawy[1,*], Mohamed E. Eissa[2], Nada A. Ashour[3], Tarek A. Yousef[2], Arwa Omar Al Khatib[4], Samia S. Hawas[5]

[1] Department of Pharmaceutical Chemistry, College of Pharmacy, The University of Mashreq, Baghdad 10023, Iraq.
[2] College of Science, Chemistry Department, Imam Mohammad Ibn Saud Islamic University, Riyadh 11623, Saudi Arabia.

[3] Department of Pharmacology and Toxicology, Faculty of Pharmacy, Mansoura National University, Gamasa 7731168, Egypt.

[4] Faculty of Pharmacy, Hourani Center for Applied Scientific Research, Al-Ahliyya Amman University, Amman, Jordan.

[5] Department of Pharmaceutical Chemistry, Faculty of Pharmacy, Horus University-Egypt, New Damietta 34518, Egypt.

* Correspondence:

Ahmed A. Al-Karmalawy: Email: akarmalawy@horus.edu.eg

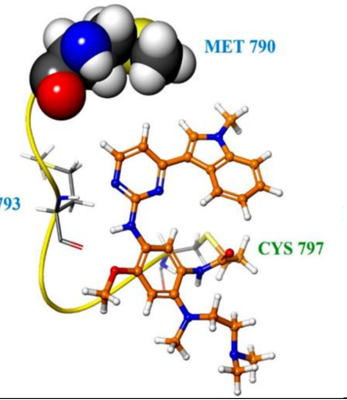

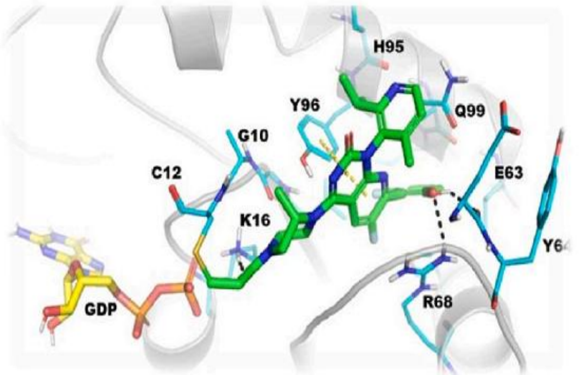

(A) Osimertenib [1]

(B) Sotorasib [2]

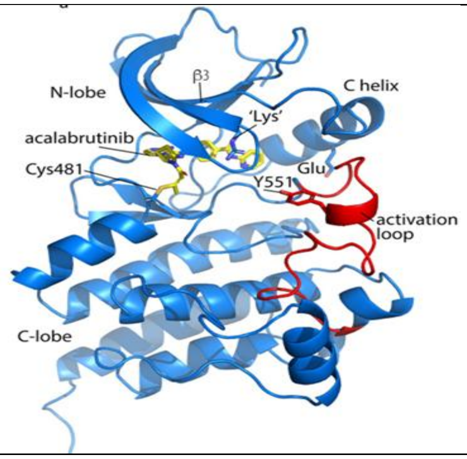

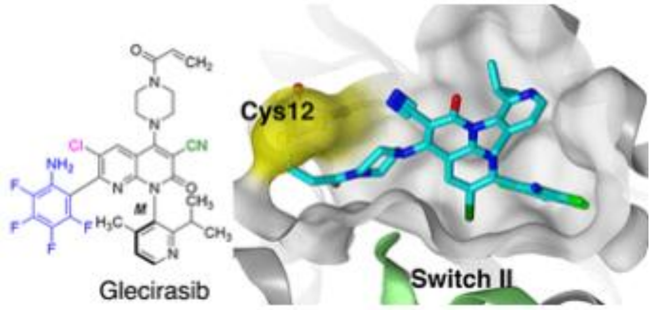

(C) Acalabrutinib [3]

(D) Glecirasib [4]

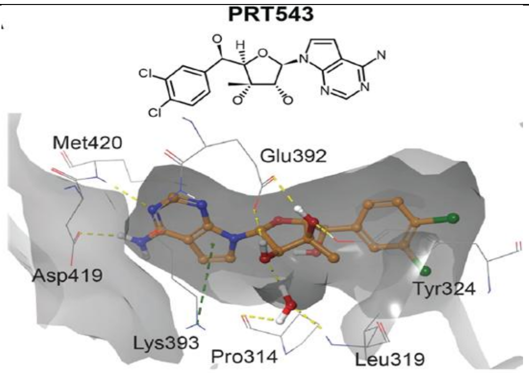

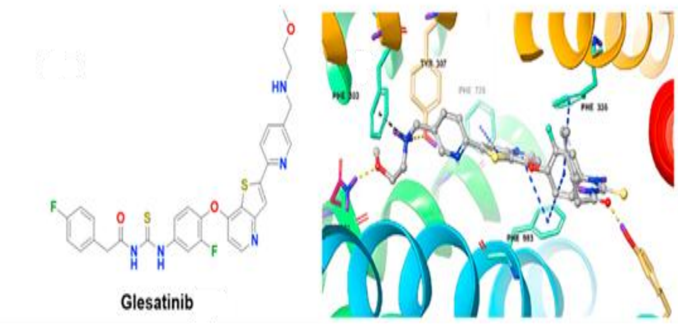

(E) PRT543 [5]

(F) Glesatinib [6]

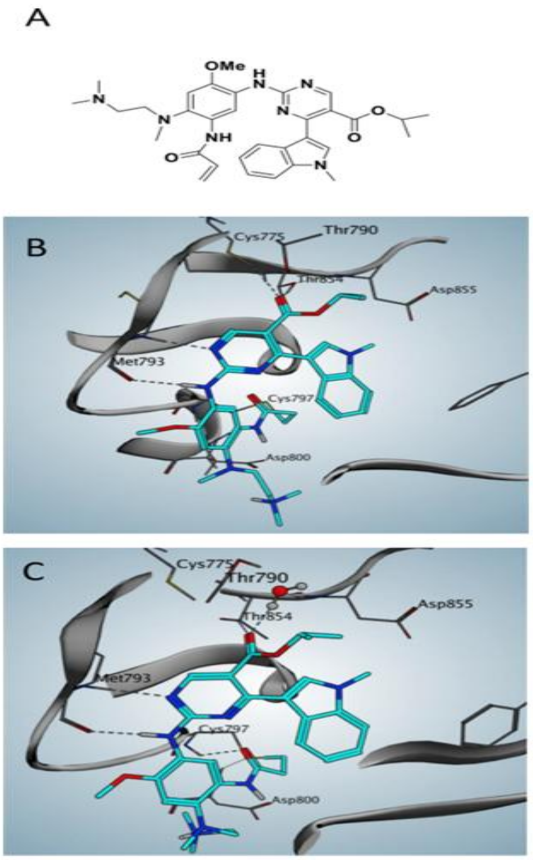

(G) Mobocertinib [7]

Fig. S1. Binding interactions of Osimertinib, Sotorasib, Acalabrutinib, Glecirasib, PRT543, Glesatinib, and Mobocertinib with the amino acid residues inside the binding pocket of the target.

References

1. B. R. Patil, K. V. Bhadane, I. Ahmad, Y. J. Agrawal, A. A. Shimpi, M. S. Dhangar, H. M. J. B. Patel and M. Chemistry, 2024, **109**, 117796.
2. D. Pandey, S. C. Chauhan, V. K. Kashyap and K. K. J. E. J. o. M. C. Roy, 2024, **277**, 116771.
3. D. Y. Lin and A. H. J. P. o. Andreotti, 2023, **18**, e0290872.
4. A. Li, S. Li, P. Wang, C. Dang, X. Fan, M. Chen, D. Liu, F. Li, H. Liu, W. Zhang, Y. Wang and Y. Wang, *Journal of Medicinal Chemistry*, 2025, **68**, 2422-2436.
5. J. P. Bewersdorf, X. Mi, B. Lu, A. Kuykendall, D. Sallman, M. Patel, D. Stevens, A. Philipovskiy, G. Sutamtewagul, L. Masarova, G. Keiffer, A. Verma, N. Bhagwat, M. Wang, A. Moore, J. Rager, D. Heiser, S. Ro, W. J. Hong, O. Abdel-Wahab and E. M. Stein, *Leukemia*, 2025, **39**, 765-769.
6. F. Jin, Y. Lin, W. Yuan, S. Wu, M. Yang, S. Ding, J. Liu and Y. Chen, *European Journal of Medicinal Chemistry*, 2024, **272**, 116477.
7. W.-S. Huang, F. Li, Y. Gong, Y. Zhang, W. Youngsaye, Y. Xu, X. Zhu, M. T. Greenfield, A. Kohlmann, P. M. J. B. Taslimi and m. c. letters, 2023, **80**, 129084.
